# Risk factors for mortality in children with hypoxemia in resource-constrained settings: a secondary analysis of Global Paediatric Acute Critical Illness Point Prevalence Study (PARITY)

**DOI:** 10.1186/s44263-025-00238-7

**Published:** 2026-01-09

**Authors:** Carter Biewen, Shän L. Ward, Asya Agulnik, Srinivas Murthy, Qalab Abbas, Adnan Bhutta, Jazmin Baez Maidana, Adrian Holloway, Jan Hau Lee, Eliana López-Barón, Christian Umuhoza, Matthew O. Wiens, Robinder G. Khemani, Teresa B. Kortz, the Global PARITY Investigators, Alhassan Abdul-Mumin, Nabisere Allen, Paloma Amarillo, Kokou Amegan-Aho, John Appiah, Pamela Arancibia, Anita Arias, Fehmina Arif, Liliana Arteaga, Jacqueline Asibey, Jonah Attebery, Nataly Ávila Guerrero, Tigist Bacha, Briam Beltran Hernandez, Hippolyte Bwiza Muhire, Juan Calderon-Cardenas, Jhon Camacho-Cruz, Mariana Lucía Cañete, Paula Caporal, Dulamragchaa Chimedbazar, Claudia Curi, Karla Emilia de Sa Rodrigues, Tenywa Emmanuel, Maria Escobar, Sofia Esposto, Arieth Figueroa Vargas, Ericka Fink, Ana Fustiñana, Marina Giulietti, Stephanie Gordon Rivera, Muhammad Irfan Habib, Pascal Havugarurema, David He, Lucia Hernandez Somerson, Nayibe Hincapie Saldarriaga, Shubhada Hooli, Jacob Isabirye, Saifullah Jamro, Juan Jaramillo-Bustamante, Liliana Jurado Salcedo, Halima Kabir, Caleb Karanja, Adama Mamby Keita, Marie-Charlyne Kilba, Niranjan Kissoon, Guillermo Kohn-Loncarica, Kandamaran Krishnamurthy, Jorhk Lasso Noguera, Marianne Majdalani, Isabel Monje Cardona, Emilse Montero Nuñez, Celia Mulgado Aguas, Raya Mussa, Fiona Muttalib, John Nebaza, Katie Nielsen, María Noya, Edna Obodai, Carmen Ocampo, Çağlar Ödek, Tagbo Oguonu, Afua Osew-Gyamfi, Sheila Owusu, Larko Owusu, Mayerly Palencia Bocarejo, Freddy Pantoja Chamorro, Aurora Pedroza, Walugembe Peter, Javier Prego, Amal Rahi, Carmen Ramírez Hernández, Kenneth Remy, Pedro Rino, Adriana Teixeira Rodrigues, Firas Sakaan, Jhuma Sankar, Hendry Sawe, Jesus Serra, Agustin Shaieb, Arianna Shirk, Enkhtur Shonkhuuz, Javier Sierra-Abaunza, Khurram Soomro, Samba Sow, Abner Tagoola, Atnafu Tekleab, Margarita Torres, Pablo Vasquez-Hoyos, Amelie von Saint Andre-von Arnim, Justin Wang, Rafiuk Yakubu, Rita Yeboah, María Zamarbideon, the Global Health Subgroup of the Pediatric Acute Lung Injury, Sepsis Investigators (PALISI) Network

**Affiliations:** 1 Division of Critical Care, Department of Pediatrics, University of California, San Francisco, San Francisco, CA USA; 2 Department of Global Pediatric Medicine, St. Jude Children’s Research Hospital, Memphis, TN USA; 3 Division of Pediatric Critical Care, Department of Pediatrics, BC Children’s Hospital, The University of British Columbia, Vancouver, BC Canada; 4 Department of Pediatrics and Child Health, Aga Khan University, Karachi, Pakistan; 5 Division of Pediatric Critical Care Medicine, Indiana University School of Medicine and Riley Children’s Health, Indianapolis, IN USA; 6 Division of Pediatric Critical Care Medicine, Department of Pediatrics, University of Maryland Baltimore, Baltimore, MD USA; 7 Children’s Intensive Care Unit, KK Women’s and Children’s Hospital, Singapore, Singapore; 8 SingHealth Duke-NUS Global Health Institute, Singapore, Singapore; 9 School of Public Health and Information Sciences, University of Louisville, Louisville, KY USA; 10 Departamento de Pediatría, Universidad de Antioquia, Medellín, Colombia; 11 Department of Pediatrics and Child Health, University of Rwanda College of Medicine and Health Sciences, Kigali, Rwanda; 12 Department of Anesthesiology, Pharmacology and Therapeutics, University of British Columbia, Vancouver, BC Canada; 13 Department of Anesthesiology and Critical Care Medicine, Children’s Hospital Los Angeles, Los Angeles, CA USA; 14 Department of Pediatrics, Keck School of Medicine, University of Southern California, Los Angeles, CA USA; 15 Institute for Global Health Sciences, University of California, San Francisco, San Francisco, CA USA

**Keywords:** Pediatrics, Global Health, Critical Illness, Hypoxemia, Resource-Constrained Setting, Pediatric Acute Respiratory Distress Syndrome (PARDS), Resource Utilization

## Abstract

**Background:**

Hypoxemia, a mortality predictor and hallmark of pediatric acute respiratory distress syndrome (PARDS), is disproportionately common in resource-constrained settings (RCS). The burden of PARDS in RCS is likely substantial considering the high prevalence of known clinical triggers (e.g., sepsis, pneumonia, trauma), but it is challenging to diagnose due to limited diagnostic resources. We aimed to: (1) describe respiratory care resource availability in RCS hospitals and test whether availability was associated with mortality; (2) determine the proportion of children who presented to RCS hospitals with hypoxemia and their associated outcomes; and (3) test whether, in children with hypoxemia, having a PARDS trigger was associated with mortality.

**Methods:**

We developed and applied operational definitions for five tiered respiratory care resource bundles. Through a secondary analysis of Global Paediatric Acute Critical Illness Point Prevalence Study (PARITY) data, we performed descriptive statistics, hypothesis testing (i.e., chi-square and Wilcoxon rank-sum tests), and logistic regression analyses.

**Results:**

Among the entire Global PARITY cohort (*n* = 7538), 763 (10.1%) were admitted with hypoxemia. Seventy percent (*n* = 531) were treated at a site with the intermediate or less respiratory care resource bundle available. Mortality was 6.8% (*n* = 52) and inversely associated with respiratory resource availability. The odds of mortality were higher for patients treated at sites with the intermediate bundle or less compared to those with the advanced or expert bundle available (adjusted odds ratio [OR] 18, 95% confidence interval [CI] 4.1–83). Fifty-six percent (*n* = 430) had a PARDS trigger, most commonly pneumonia (*n* = 256), bronchiolitis (*n* = 116), and sepsis (*n* = 58). There was no association between the presence of a PARDS trigger and mortality. Ninety-four percent of patients with a PARDS trigger (*n* = 405/430) had insufficient data available for a PARDS-related diagnosis according to the Second Pediatric Acute Lung Injury Consensus Conference (PALICC-2) guidelines.

**Conclusions:**

Children with hypoxemia treated at hospitals with respiratory care resource constraints in countries with lower socio-demographic index (SDI) had significantly higher mortality. These findings highlight the importance of ongoing work to improve resource availability, strengthen health systems, and support pediatric healthcare providers in identifying PARDS in order to help clinicians risk stratify children, focus resources, and tailor management to optimize outcomes.

**Supplementary Information:**

The online version contains supplementary material available at 10.1186/s44263-025-00238-7.

## Background

Hypoxemia is common in children; the World Health Organization (WHO) reported a 31% prevalence of hypoxemia among children with WHO-classified pneumonia worldwide [1, 2]. Hypoxemia is a known risk factor for mortality in certain pediatric disease states such as pneumonia and malaria [3–5]. In resource-constrained settings (RCS), where a lack of funds results in limited access to medications, equipment, and supplemental oxygen, less-developed infrastructure, and fewer or less-trained personnel [6, 7], hypoxemia is likely underappreciated, and mortality associated with hypoxemia is likely disproportionately high. In a recent survey of 238 hospitals in 60 countries, only 85% of intensive care units (ICUs) in low- and low-middle-income countries reported consistent availability of basic respiratory support (e.g., oxygen, suction, nebulizer) [6]. There is a suggested association between lack of resources and worse outcomes; for example, higher case fatality rates from lower respiratory tract infection (LRTI) have been observed in low- versus middle-income countries [8]. To date, limited pediatric studies in RCS have evaluated the association between respiratory care resource availability and mortality.

LRTI is a common cause of hypoxemia in children and the leading cause of under-5 mortality worldwide outside of the neonatal period, with an estimated 700,000 deaths globally in 2022 [9]. Pediatric acute respiratory distress syndrome (PARDS), a disorder of acute lung inflammation characterized by hypoxemia, can occur following severe LRTI and other acute triggers such as sepsis, trauma, aspiration, drowning, and shock. PARDS is associated with high mortality that requires specialized care for diagnosis and management [10–12]. In children admitted to the pediatric intensive care unit (PICU) with PARDS, mortality is higher in middle-income compared with high-income countries [13, 14]. Little is known about PARDS in RCS where diagnostic resources may be lacking (e.g., pulse oximetry, chest X-ray, invasive and non-invasive positive pressure ventilation, blood gas analysis) [15]; however, considering the high prevalence of clinical conditions known to trigger PARDS (e.g., sepsis, pneumonia, trauma), the morbidity and mortality of PARDS in RCS is suspected to be significant.

An improved understanding of risk factors associated with poor outcomes in children with hypoxemia, and resources available to care for children with respiratory disease, will facilitate further work to identify and optimize the management of children with acute hypoxemia in RCS. We hypothesized that decreased respiratory care resource availability was associated with increased mortality in children with hypoxemia presenting to RCS hospitals. Additionally, given resource constraints preventing accurate diagnosis of PARDS in these settings, we hypothesized that the presence of a PARDS trigger in children with hypoxemia, as a proxy for PARDS, was associated with increased mortality. The objectives of this study were to: (1) describe respiratory care resource availability in participating RCS hospitals and test whether it was associated with mortality among children with hypoxemia; (2) determine the proportion of children presenting to hospitals in RCS with hypoxemia and their associated outcomes; and (3) test whether, in children with hypoxemia, having a PARDS trigger was associated with mortality.

## Methods

### Global Paediatric Acute Critical Illness Point Prevalence Study (PARITY) dataset

We performed a secondary analysis of the data from Global PARITY [16], which was an international, prospective point prevalence study aimed at estimating the burden of pediatric acute critical illness in RCS. The study was conducted during four sampling time points to capture seasonal variation over a 12-month period and enrolled 7538 patients across 46 sites in 19 countries (Supplementary Material 1: Fig. S1). The Global PARITY population included children ages 29 days to 14 years evaluated for acute illness or injury in the emergency department (and subsequently discharged or admitted to the hospital) or directly admitted to an inpatient unit. Admitted children were assessed daily for clinical status and resource utilization over the initial 7 days of admission and followed up to hospital day 30 for outcomes. In addition to case report forms for individual patients, participating sites reported hospital-level resource availability using a separate infrastructure and resource survey (Supplementary Material 1). This survey was most often completed by consultant physicians working at each site [6]. We adhered to a fully descriptive analytical approach, avoiding imputation, extrapolation, or modeling beyond the directly observed data. We followed the STROBE (Strengthening the Reporting of Observational Studies in Epidemiology) reporting guidelines. [17] (Supplementary Material 1: Table S1).

### Participating sites

Site-level hospital data included setting type (e.g., rural, urban), funding source (e.g., public, private, mixed), academic center designation (e.g. medical school affiliation), average daily hospital census, presence of a PICU, and pediatric intensivist availability. We summarized site-level data by socio-demographic index (SDI), a composite indicator of country development status that accounts for income, education, and fertility and is strongly correlated with health outcomes [7]. Only the four lowest SDI quintiles were represented (low, low-middle, middle, and high-middle). We also summarized sites by region according to the Global Burden of Disease Report, in which countries were grouped based on geographic proximity, epidemiologic similarity, and cause of death distribution similarity [18].

### Respiratory care resources

**Fig. 1:**
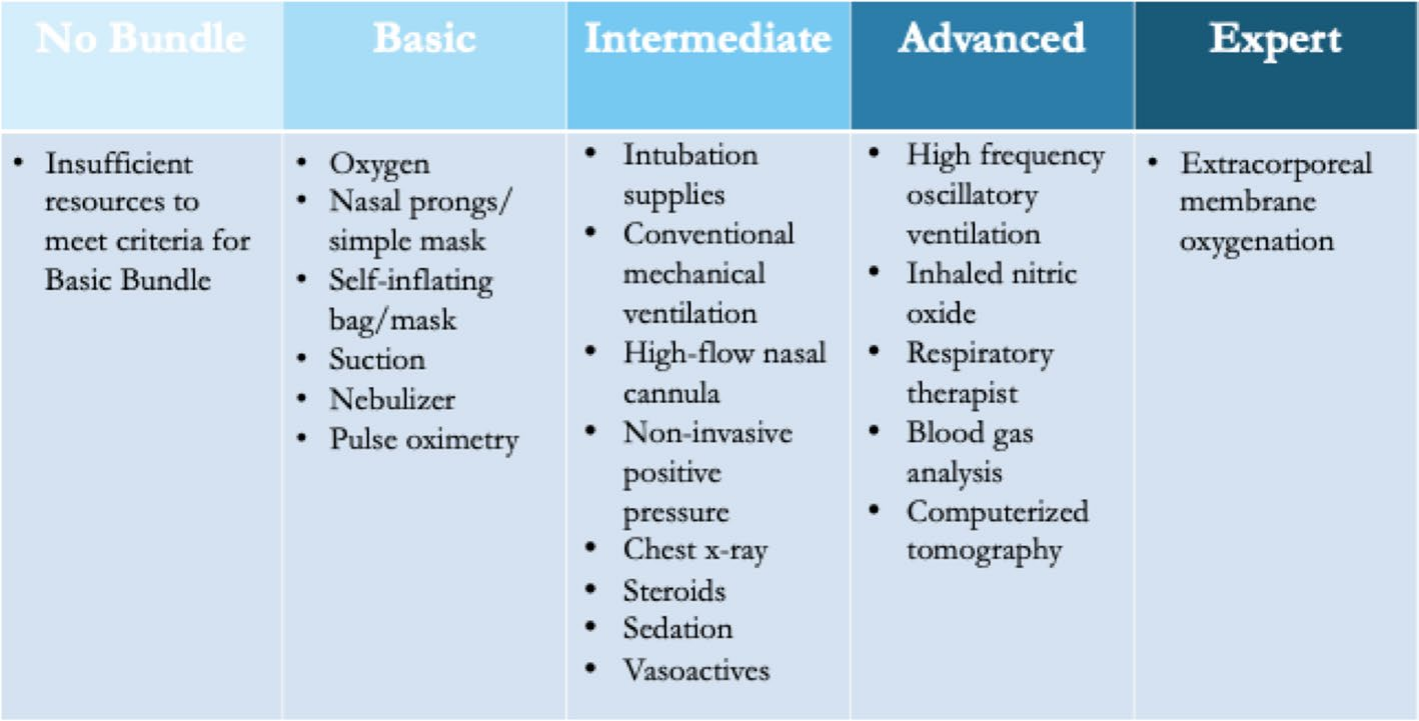
Respiratory care resource bundles

We determined essential respiratory care resources and created operational definitions of tiered resource bundles (categorized as one of five bundles from no bundle through expert*)* informed by Ferrari et al. [18] and in collaboration with a group of international experts developing a pediatric critical care resource evaluation tool for RCS [19, 20] (Fig. 1).

We summarized respiratory care resource availability at each site by analyzing responses to the Global PARITY Pediatric Acute Care Infrastructure and Resource Availability Survey (Supplementary Material 1), which asked about the availability of all resources included in the bundles. A resource bundle was considered available at a site if at least 80% of the individual resources included in that bundle were reported to be “always” or “often” available on a 5-point Likert scale. We reported the highest resource bundle available at each site; all bundles at or below the level reported were also available (e.g., sites with the advanced bundle available also had at least 80% of intermediate and basic bundle resources available). We summarized resource bundle availability by patient, site, and SDI. Pulse oximetry was defined as either portable or continuous oximetry. Intubation supplies were defined as laryngoscopes and endotracheal tubes. Sedation was defined as any of intravenous opioids, benzodiazepines, propofol, or ketamine.

We reported resources utilized by patients during the first 7 days of hospitalization as reported on the Global PARITY case report form. This included the highest level of respiratory support utilized by the patient (i.e., invasive mechanical ventilation, continuous positive airway pressure [CPAP], bilevel positive airway pressure [BiPAP], high-flow nasal cannula (HFNC), simple or low flow oxygen, none of these), whether a chest X-ray was obtained, if the patient required continuous sedation longer than 4 h, and whether the patient required vasoactive medications (e.g., epinephrine, norepinephrine, dopamine).

### Patient population and data

In this secondary analysis of Global PARITY data [16], we included only children with hypoxemia, defined as those having an oxygen saturation < 88% and/or receiving any form of supplemental oxygen within the first 7 days of hospital admission, or experiencing death in the emergency department with an oxygen saturation < 88% or while receiving supplemental oxygen. This oxygenation threshold of <88% is consistent with the threshold used to define At-risk for PARDS from the Second Pediatric Acute Lung Injury Consensus Conference (PALICC-2) [10]. We excluded patients with cyanotic heart disease (e.g., tetralogy of Fallot, truncus arteriosus, transposition of the great arteries, total or partial anomalous venous drainage, tricuspid atresia, undifferentiated “cyanotic” heart disease), as we could not determine baseline oxygen saturations.

Patient-level data included demographics, comorbidities (e.g., malnutrition), Lambaréné Organ Dysfunction Score (LODS) on admission, PARDS diagnostic criteria (e.g., chest X-ray result, PARDS trigger, highest level of respiratory support, oxygen saturation, mean airway pressure 6 h after starting mechanical ventilation, fraction of inspired oxygen 6 h after starting mechanical ventilation), and final diagnoses. Malnutrition was considered present if the mid-upper arm circumference was less than 12.5 cm, the weight-for-age Z-score was < −2, or comorbid malnutrition was reported on the initial intake form. The LODS, a simple mortality prediction score developed and validated in malaria endemic regions [21], was used as a marker of the severity of illness and calculated on a three-point scale using the presence of prostration (or inability to feed if <5 months), coma (Blantyre Coma Score < 3, Glasgow Coma Score ≤ 8 or reported unconscious), and deep breathing. The primary outcome was in-hospital mortality. The secondary outcome was hospital length of stay (LOS) among survivors.

Patients were categorized into two groups: those with a PARDS trigger, defined as a final diagnosis of pneumonia, bronchiolitis [22], coronavirus disease of 2019 (COVID-19), sepsis, trauma, aspiration, drowning, or other shock [13], and those without a reported PARDS trigger (e.g., gastroenteritis, acute malaria, meningitis/encephalitis, status epilepticus, asthma). Patients with pneumonia, bronchiolitis, and/or COVID-19 were collectively categorized as LRTI. Patients with sepsis and trauma were analyzed independently. Patients with aspiration, drowning, or other shock were combined to comprise an “other” PARDS trigger group for analysis due to a limited number of patients with these diagnoses.

PARDS diagnosis and severity were determined according to criteria from PALICC-2 [23] Supplementary Material 1: Table S2). Due to a lack of blood gas data, the saturation/fraction of the inspired oxygen ratio was used to determine oxygenation impairment. Children with a PARDS trigger, as listed above, were then categorized as having PARDS, Possible PARDS, or At-risk for PARDS. PARDS criteria included all of the following: an opacity on chest X-ray, the need for positive pressure ventilation, and meeting oxygenation criteria. Airway pressure data were not captured, so we were unable to determine if patients receiving non-invasive positive pressure ventilation met PARDS criteria. In patients with an oxygen saturation > 97%, we were unable to use the oxygenation criteria for PARDS diagnosis as the oxyhemoglobin dissociation curve flattens above 97% [24]. Possible PARDS criteria included the need for nasal respiratory support (positive pressure or HFNC) and meeting oxygenation criteria in the absence of chest X-ray results. At-risk for PARDS criteria included an opacity on chest x-ray and the need for oxygen supplementation via any interface, without satisfying the criteria for PARDS or Possible PARDS. Minimum acceptable liter flow thresholds for Possible PARDS and At-risk for PARDS diagnosis were established in accordance with PALICC-2 [23].

We assumed that patients did not have perinatal lung disease due to the study inclusion criteria (>28 days), and the fact that all were admitted from home with no documentation of chronic respiratory conditions in the case report forms. Chronic lung disease was considered unlikely in patients without pre-existing pulmonary diagnoses documented in the case report forms at the time of admission. If diagnosed with a PARDS trigger, we assumed that they were within 7 days of the clinical insult having presented to the hospital acutely. Case report forms indicated the presence, location, and interpretation of chest imaging opacification; the case report form did not specify by whom the interpretation was provided (i.e., clinician or radiologist). When interpretation assessing whether opacities were due to atelectasis, cardiac failure, or fluid overload was not available, we assumed that an opacification represented acute pulmonary parenchymal disease. To assess oxygenation, we used the data collected for mean airway pressure, fraction of inspired oxygen, and oxygen saturation documented 6 hours after starting mechanical ventilation.

### Statistical analysis

All statistical analyses were performed using Stata 17.0 (StataCorp LLC, College Station, TX, USA) [25]. We performed descriptive statistics to summarize site-, resource-, and patient-level data, which are presented as frequencies (percentage), means (standard deviation, SD), or medians (interquartile range, IQR) as appropriate given the underlying data and data distribution.

We compared differences between groups (e.g., PARDS trigger vs. no PARDS trigger, survivors vs. non-survivors) using chi-square tests for categorical variables and Wilcoxon rank-sum tests for continuous variables. A p-value < 0.05 was considered statistically significant.

To test whether respiratory care resource bundle availability was associated with mortality, we performed univariate and multivariable logistic regression modeling controlling for confounders – average daily hospital census (>50 patients), PICU availability, and academic center designation. Variables were determined a priori based on our hypotheses, literature review, and the directed acyclic graph (Supplementary Material 1: Fig. S2). Variables were tested for collinearity, and in the case of variables being highly correlated, were down selected for inclusion in the final model. For these analyses, we categorized patients into two resource bundle groups based on mortality trends (intermediate or less, and advanced or expert).

To test whether the presence of a PARDS trigger was associated with mortality, we performed univariate and multivariable logistic regression modeling controlling for confounders: age (≤5 years), presence of malnutrition, LODS (>0), and site respiratory care resource availability (two groups as above). Confounders were determined a priori based on our hypotheses (Supplementary Material 1: Fig. S3) and a review of the literature [8, 21, 26, 27]. We reported unadjusted and adjusted odds ratios (OR) with 95% confidence intervals (CI).

## Results

### Sites

Of the 46 participating Global PARITY sites, most were located in Sub-Saharan Africa (*n* = 18 sites, 39%) and Latin America/Caribbean (*n* = 14 sites, 30%). Sites were evenly distributed between the four lowest SDI quintiles: low SDI (*n* = 10 sites, 22%), low-middle SDI (*n* = 13 sites, 28%), middle SDI (*n* = 13 sites, 28%), and high-middle SDI (*n* = 10 sites, 22%). Eighty-nine percent (*n* = 41 sites) were in an urban setting and 72% (*n* = 33 sites) were designated academic centers. Thirty-nine percent (*n* = 18 sites) had an average daily pediatric census of greater than 50 patients. Sixty-five percent (*n* = 30 sites) reported a PICU available, and 54% (*n* = 25 sites) reported a pediatric intensivist available during the weekday in the hospital.

#### Respiratory care resources

Three sites (6.5%) did not meet the minimal requirements of the basic respiratory care resource bundle and were categorized as having no bundle available. Eleven sites (24%) had the basic bundle, 13 sites (28%) had the intermediate bundle, 16 sites (35%) had the advanced bundle, and three sites (6.5%) had the expert bundle available. Of all Global Burden of Disease regions, Sub-Saharan Africa had the highest proportion of sites with less than the intermediate resource bundle available (56%). South Asia had the highest proportion of sites with greater than the intermediate resource bundle available (72%). In general, respiratory care resource availability increased with increasing SDI; 17 of 19 sites (89%) with the advanced or expert resource bundle available were located in middle or high-middle SDI countries (Supplementary Material 1: Fig. S4). In low- and low-middle SDI countries, HFNC and non-invasive positive pressure ventilation were each available at 13 sites (57%) and conventional mechanical ventilation at 14 of 23 sites (60%) (Supplementary Material 1: Table S3).

#### Patient characteristics

**Fig. 2:**
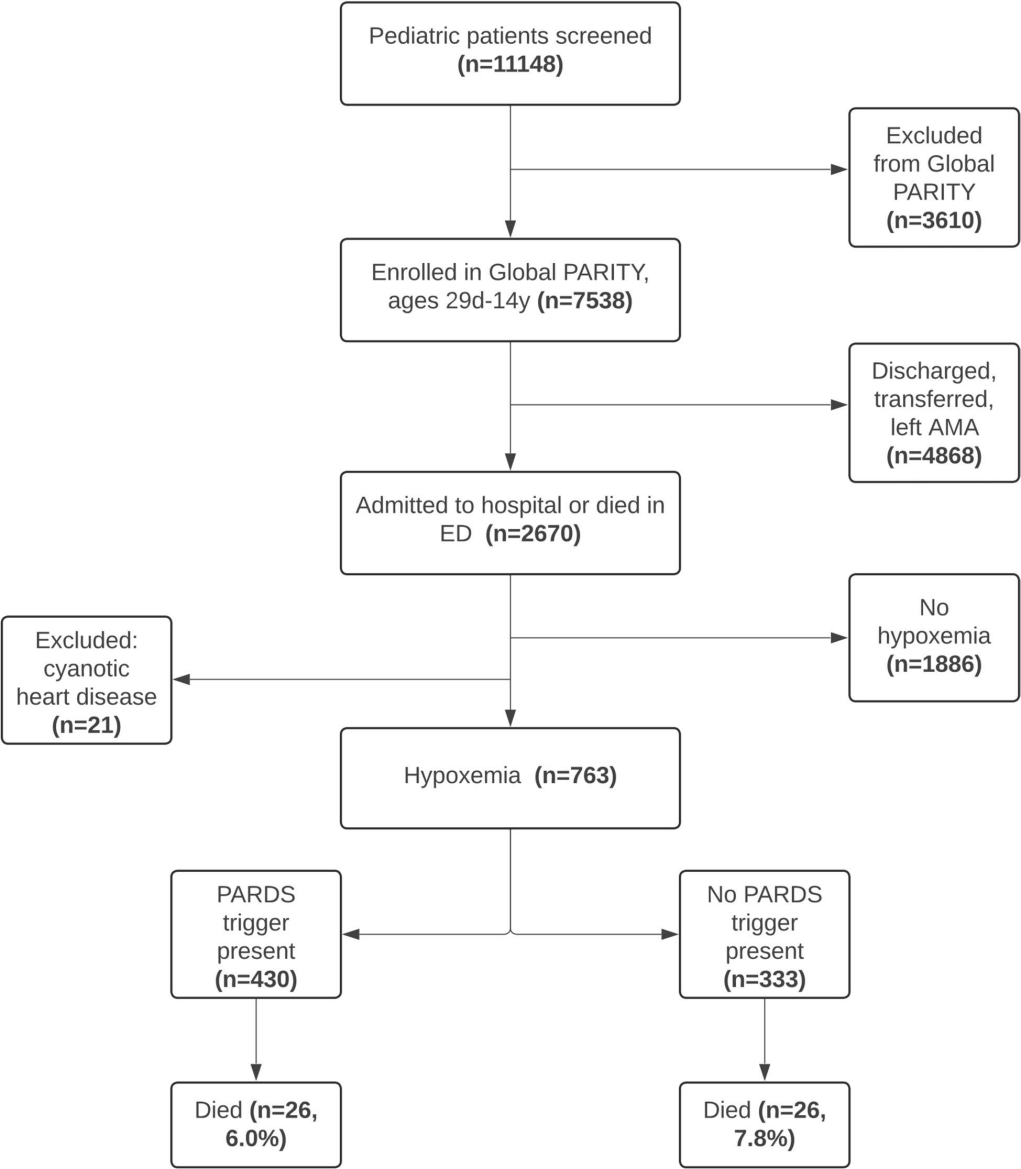
Patient screening and inclusion. Legend: AMA,   against medical advice; ED,  emergency department; PARDS,  pediatric acute respiratory distress syndrome

Of the entire Global PARITY cohort, 763 of 7538 patients (10.1%) experienced hypoxemia while hospitalized and were included in this secondary analysis (Fig. 2). Characteristics of children with hypoxemia are presented in Table 1. The median age was 1.9 years (IQR 0.6-5years) and 44% (*n* = 336 patients) were female. Forty-five percent (*n* = 343 patients) were treated at a site in a low SDI quintile country. Seventy percent (*n* = 531 patients) were treated at a site with the intermediate respiratory care resource bundle or less available. Of 430 children with hypoxemia and a PARDS trigger, the most common triggers were pneumonia (*n* = 256 patients, 60%), bronchiolitis (*n* = 116 patients, 27%), and sepsis (*n* = 58 patients, 13%). Compared to children without a PARDS trigger, children with a PARDS trigger were younger, had lower oxygen saturation, and higher LODS on admission (Table 1).

**Table 1:** Characteristics of hypoxemic children with or without a PARDS trigger admitted to resource-constrained hospitals

	**Characteristics**				
	**Total (*n* = 763)**	**PARDS trigger (*n* = 430)**	**No PARDS trigger (*n* = 333)**	**Missing data, *n* (%)**	***p*-value**
Age (years), median (IQR)	1.9 (0.6–5)	1 (0.4–3)	3 (1.2–7)	**-**	** <0.001**
Female, n(%)	336 (44)	190 (44)	146 (44)	1 (0.1)	0.5
Weight (kilograms), median (IQR)	10 (6.5–16)	8.4 (5.7–13)	13.3 (8.7–20)	77 (10)	** <0.001**
SpO2 (%) on admission, median (IQR)	95 (91–98)	94 (89–98)	96 (92–98)	35(4.6)	** <0.001**
Malnutrition, *n* (%)	196 (26)	114 (27)	82 (25)	-	0.55
LODS, median (IQR)	1 (0–1)	1 (0–1)	0 (0–1)	-	** <0.001**
Global Burden of Disease Region				-	0.14
SSA, *n* (%)	267(35)	156 (36)	111 (33)		
SA, *n* (%)	222 (29)	119 (28)	103 (31)		
LA, *n* (%)	169 (22)	92 (21)	77 (23)		
CE, *n* (%)	22 (2.9)	10 (2.3)	12 (3.6)		
NA, *n* (%)	15 (2.0)	13 (3.0)	2 (0.6)		
SLA, *n* (%)	68 (8.9)	40 (9.3)	28 (8.4)		
Socio-demographic Index				-	0.08
Low, *n* (%)	343(45)	202 (47)	141 (42)		
Low-Middle, *n *(%)	168 (22)	83 (19)	85 (26)		
Middle, *n *(%)	168 (22)	91 (21)	77 (23)		
High-Middle, *n *(%)	84 (11)	54 (13)	30 (9.0)		

*P* values <0.05 represent a significant difference in characteristic distribution between groups, calculated with a chi-square test (categorical variables) or Wilcoxon rank-sum test (continuous variables)

*PARDS* Pediatric acute respiratory distress syndrome, *IQR *Interquartile range, *SpO2 *Oxygen saturation, *LODS *Lambaréné Organ Dysfunction Score, *CE *Central Europe, Eastern Europe, and Central Asia, *LA *Latin America and Caribbean, *SLA *Southern Latin America, *NA *North Africa and Middle East, *SA *South Asia, *SSA *Sub-Saharan Africa

**Fig. 3:**
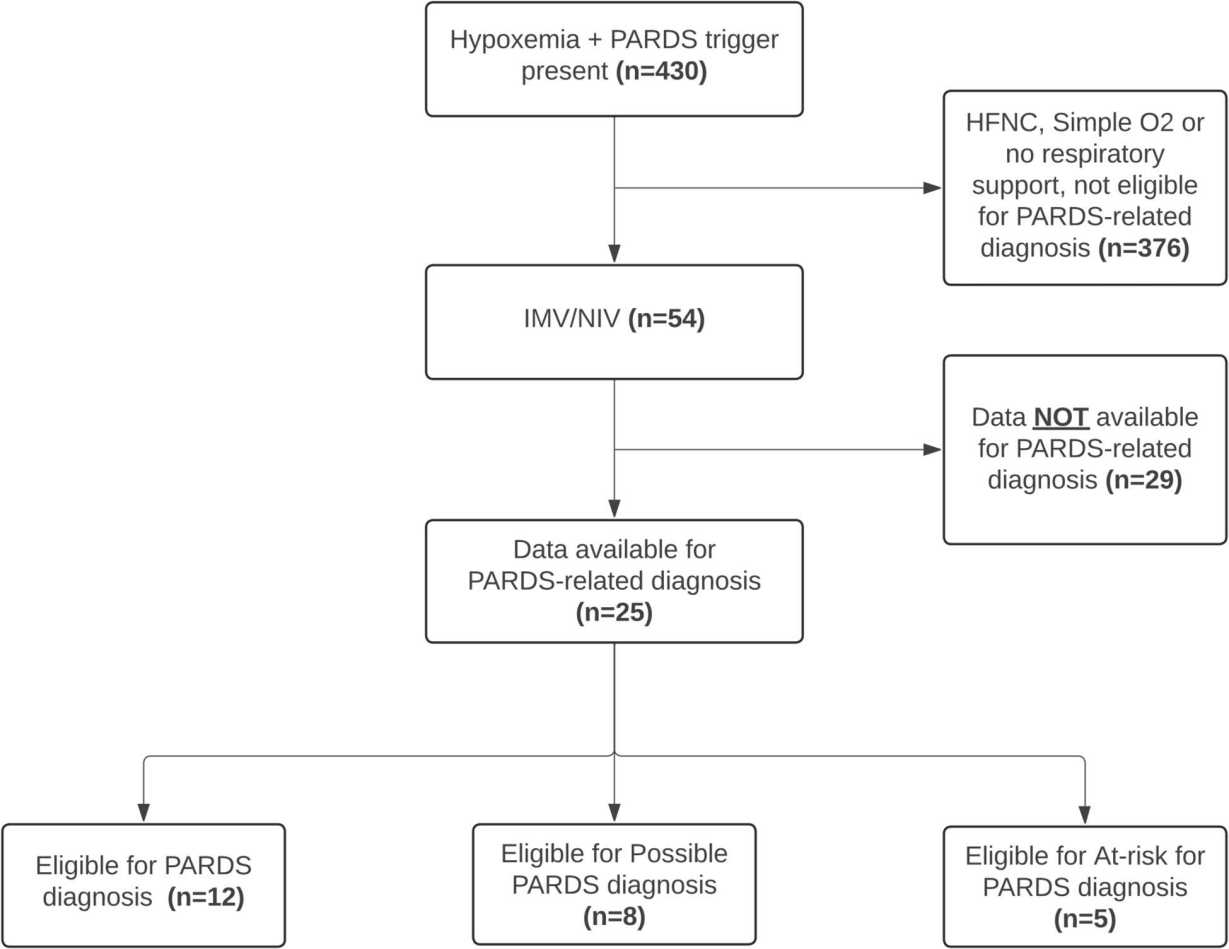
Patient eligibility (i.e., sufficient data available) for PARDS-related diagnoses (PARDS, Possible PARDS, At-risk for PARDS). Legend: PARDS,  pediatric acute respiratory distress syndrome; IMV,  invasive mechanical ventilation; NIV,  non-invasive ventilation; HFNC,  high-flow nasal cannula; O2,  oxygen

Respiratory resource utilization was similar between those with and without a PARDS trigger (Supplementary Material 1: Table S4); 369/430 patients (86%) with a PARDS trigger versus 283/333 patients (85%) without a PARDS trigger received HFNC or simple O2, and 54/430 patients (13%) with a PARDS trigger versus 41/333 patients (12%) without a PARDS trigger received invasive or non-invasive mechanical ventilation. Only 25 patients (5.8%) had sufficient data to consider any PARDS-related diagnosis (Fig. 3). The most commonly missing data required to make a PARDS diagnosis were fraction of inspired oxygen, mean airway pressure, and type of non-invasive interface for children receiving mechanical ventilation (Supplementary Material 1: Table S5); these data were more likely to be missing from sites in lower SDI quintile countries (Supplementary Material 1: Table S6).

Five of 12 patients (42%) with sufficient data for a diagnosis of PARDS met PARDS criteria. Three of eight eligible patients (38%) met criteria for Possible PARDS. Of the remaining 14 patients with sufficient data who did not meet PARDS or Possible PARDS criteria, six patients (43%) met At-risk for PARDS criteria according to PALICC-2 guidelines (Supplementary Material 1: Fig. S5).

#### Outcomes

In-hospital mortality among children with hypoxemia was 6.8% (*n* = 52/763 patients) compared to 0.8% (*n* = 16/1886 patients) for those admitted to the hospital without hypoxemia (*p* < 0.001). Among children admitted with hypoxemia, those who died were older (median 3.5 years [interquartile range (IQR) 0.5–4] vs. 1.9 years [IQR 0.7–7.5], *p* = 0.02) and more likely to be malnourished (*n* = 21/52 patients [40%] vs. *n* = 176/711 patients [25%], *p* = 0.01). Most patients were admitted in a country with low- or low-middle SDI (*n* = 49/52 patients, 94%), and the highest mortality was observed in Sub-Saharan Africa (*n* = 33/267 patients, 12%) (Supplementary Material 1: Table S7). Simple oxygen supplementation was the most common respiratory resource utilized by children with hypoxemia in both the survivors and non-survivors (*n* = 530/711 patients [75%] and *n* = 23/52 patients [44%], respectively) (Supplementary Material 1: Table S8).

**Fig. 4:**
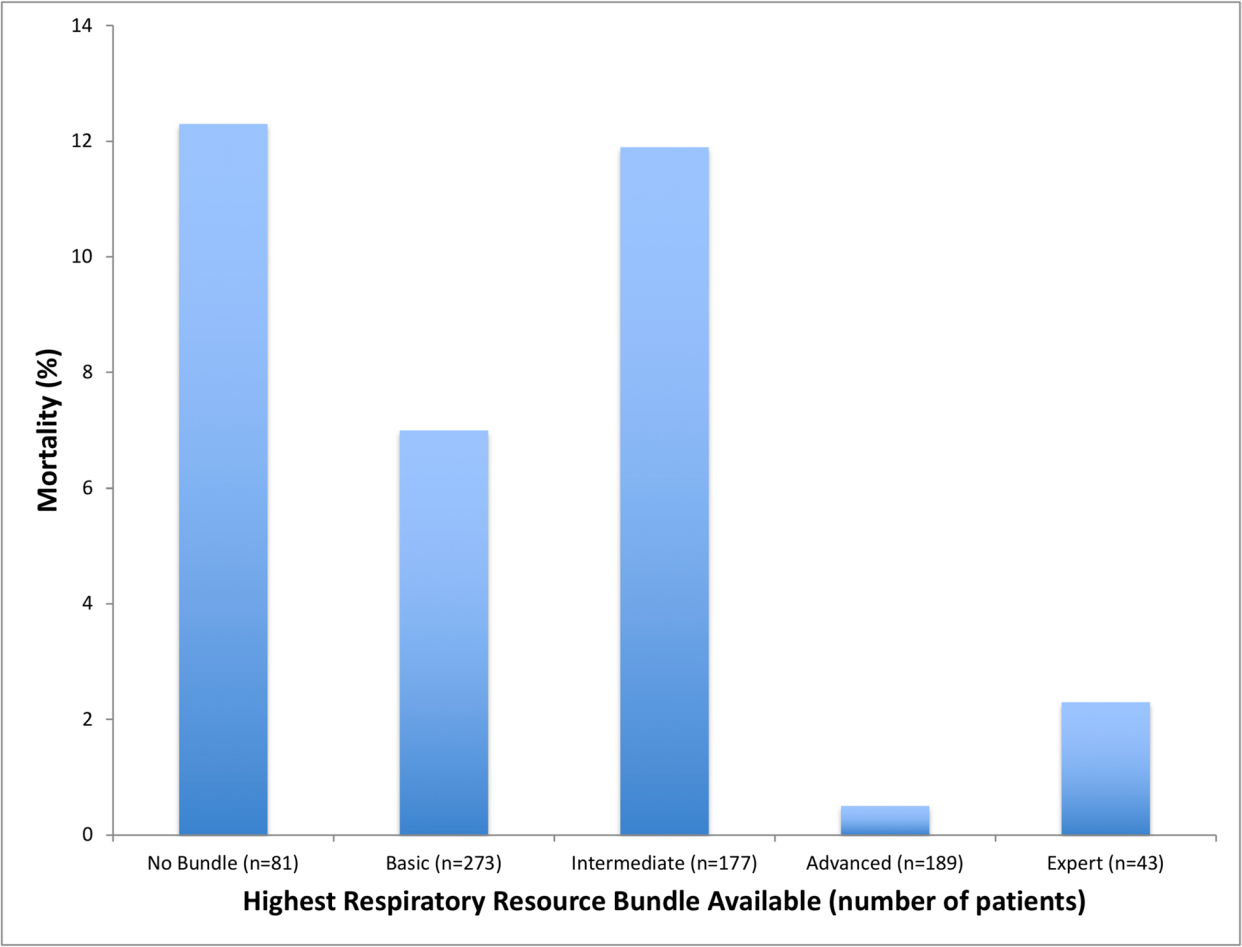
Mortality by respiratory care resource bundle availability. *n = total number of patients with hypoxemia treated at sites with each resource bundle available

Mortality differed by respiratory care resource bundle availability with the highest mortality at the sites with no bundle (*n* = 10/81 patients [12%]), the intermediate (*n* = 21/177 patients [12%]), and the basic bundles (*n* = 19/273 patients [7%]), as compared to the advanced (*n* = 1/189 patients [0.5%]) and expert (*n* = 1/43 patients [2.3%], *p* < 0.001) bundles (Fig. 4). Patients treated at sites with the intermediate, basic, or no bundles had higher odds of mortality compared to those treated at sites with the advanced or expert bundles (OR 18, 95% CI 4.1–83) when adjusted for average daily hospital census, PICU availability, and academic center designation (Supplementary Material 1: Table S9).

The presence of a PARDS trigger was not associated with mortality. Twenty-six of 430 patients with a PARDS trigger died (6%). The unadjusted OR for mortality in patients with a PARDS trigger compared to patients without a PARDS trigger was 0.76 (95% CI 0.43–1.3) and the adjusted OR was 0.56 (95% CI 0.30–1.04; adjusted for resource availability, age, malnutrition, and LODS) (Supplementary Material 1: Table S10).

The median hospital LOS among survivors was 3.25 days (IQR 1–6.6 days). Median hospital LOS among survivors was longer for children with a PARDS trigger compared to those with no PARDS trigger (median 4 days [IQR 1.8–7] vs. 2.6 days [IQR 1–5.8], *p* < 0.001).

## Discussion

We found that hypoxemia was common (10%) in children presenting to RCS hospitals, 70% of children with hypoxemia were treated at sites with an intermediate or less respiratory care resource bundle, and mortality was inversely associated with both respiratory care resource availability and SDI. This is consistent with prior studies demonstrating higher mortality with decreasing country development level [8, 13, 26]. Only two of 232 (<1%) hypoxemic patients died when the advanced or expert respiratory care resource bundle was available compared with 50 of 531 (9.4%) patients when the intermediate bundle or less were available. These findings suggest that hospitals with resource constraints care for a high burden of respiratory illness and have higher associated mortality; this highlights the urgent need to optimize resources to ensure all hospitals can provide appropriate respiratory care to children with hypoxemia. Furthermore, it is important to highlight that 90% of sites with advanced or expert respiratory care resource bundles available were located in middle- or high-middle SDI countries where basic systems and infrastructure may also be more robust. While increasing advanced respiratory care resources has the potential to improve pediatric outcomes, it is first critical to ensure basic care is available and provided well. Despite consensus recommendations from the WHO for the management of hypoxemia, there exists heterogeneity in the identification and management of hypoxemia due to resource constraints, cost, practice variation, and lack of standardized protocols [2, 15, 28–30]. Successfully caring for children with hypoxemia relies on timely recognition, sufficient resources, provider training in the management of pediatric respiratory illness, and coordination of a complex healthcare system. Policies and funding should support interventions focused on these areas and be implemented thoughtfully based on local healthcare systems, disease burden, and resource needs [6, 16].

This secondary analysis also illustrated the significant burden of hypoxemia in RCS, both with respect to prevalence (10%) and associated inpatient mortality (6.8%). This is consistent with hypoxemia prevalence previously reported in a pediatric cohort admitted to hospitals in southwest Nigeria (10.2%) [1], as well as a pediatric cohort with non-severe pneumonia in low- and middle-income country hospitals (8%) [1, 2]. The 6.8% mortality from this study was higher than the reported case fatality rate of children admitted with hypoxemia in southwest Nigeria (3.4–5.6%) [1] and fell between the 28-day mortality of children hospitalized in Uganda and Kenya with pneumonia and hypoxemia (~4%) or severe hypoxemia (~20%) who received no support, low- or high-flow nasal cannula [31]. We believe that the heterogeneity in site resource availability in this study contributed to the lower observed mortality as compared to previously published estimates in children with severe hypoxemia from single countries.

Through this secondary analysis, we gained insight into the applicability of PALICC-2 criteria in RCS, finding that 94% of children with hypoxemia and a PARDS trigger receiving respiratory support had insufficient data available to make a PARDS-related diagnosis. PALICC-2 criteria were designed to be more inclusive of resource-variable settings and were used to inform the Global PARITY data collection form; however, missing data limited the ability to diagnose Possible PARDS and risk-stratify children with hypoxemia in this cohort. Given the challenges associated with identifying PARDS, it would be valuable to further explore the underlying reasons for missing data. Importantly, a diagnosis of PARDS, Possible, or At-risk for PARDS was limited by lack of mean airway and positive pressure settings and liter flow data, which were not collected despite being fields on the case report form for those receiving non-invasive ventilation, HFNC, or simple oxygen. Hospitals in lower SDI countries may face greater challenges with clinical data collection, documentation, measurement, and/or electronic data entry, all of which could be influenced by resource availability and systemic factors. This study did not provide funding for research staff, and prior work has demonstrated that human resource shortages contribute to challenges with vital sign measurement and documentation [32]. Furthermore, it is plausible that centers with extreme resource constraints may lack the diagnostic capacity (e.g., chest imaging), clinical infrastructure (e.g., advanced ventilator support), and trained personnel necessary to systematically and efficiently confirm PARDS according to PALICC-2 criteria; this may contribute to under-recognition or misclassification of PARDS in resource-constrained settings. Future research could examine how these systemic limitations influence the epidemiology of PARDS.

As PARDS remains an elusive diagnosis in RCS, we hypothesized that the presence of a PARDS trigger would be associated with mortality; however, we did not find this to be the case. This analysis was likely underpowered to detect an association given the overall low number of mortality events. Additionally, PARDS triggers may have been underappreciated; for example, sepsis is a difficult diagnosis to make accurately in resource-constrained settings. Another possible explanation for this lack of association is that children without a PARDS trigger may have had a similar degree of critical illness with multi-system disease (e.g., malaria) leading to hypoxemia, and other variables (e.g., malnutrition and resource availability) were more influential than lung injury in determining clinical outcomes in RCS. Cornerstones of PARDS management include the identification of hypoxemia, oxygen therapy, followed by lung protective respiratory support including non-invasive and invasive mechanical ventilation; children with “possible PARDS” should ideally be managed as if they have PARDS [10]. Given these proven beneficial management strategies, a crucial step in improving outcomes of children with PARDS in RCS is having the ability to make a timely PARDS diagnosis. Just as critical is the identification of children with Possible PARDS or At-risk for PARDS to facilitate the implementation of strategies to prevent lung disease progression and clinical deterioration. For these reasons, pulse oximetry, organized clinical data collection, and improved documentation should be prioritized in RCS.

We acknowledge several limitations related to data availability and the inherent constraints of secondary analyses. While we found an association between mortality and respiratory care resource constraints, we were unable to determine if deaths were directly attributable to respiratory disease or preventable with improved respiratory care resources. However, given that hypoxemia is a strong predictor of mortality and a driver of organ failure [2], it is reasonable to infer that the burden of mortality and organ dysfunction could have been mitigated with better respiratory support and oxygen delivery. Though we could not account for all confounders in RCS where hospitals may be hindered by challenges beyond limited respiratory care resources (e.g., other resource infrastructure, sanitation practices, safety protocols, staff training), our findings demonstrate important resource limitations in areas where hypoxemia is common and hypoxemia-related deaths are high. Additionally, we likely did not capture all patients with hypoxemia. The exclusion of children with reported cyanotic heart disease, many of whom were diagnosed with LRTI or sepsis, may have led to an underestimation of mortality, as we would expect these children to be at higher risk of death from infections. Additionally, we did not capture patients who developed hypoxemia after day 7 of hospital admission, after which, daily data were not collected. Late-onset hypoxemia is more likely to represent complications of hospitalization (e.g., hospital-acquired pneumonia and sepsis), though the exclusion of these patients may have underestimated the PARDS trigger subgroup and associated mortality. Conversely, there were limitations that likely overestimated the hypoxemia cohort size. In an attempt to capture all patients with hypoxemia, we included any child who received supplemental oxygen; however, the use of supplemental oxygen does not necessarily correspond with hypoxemia; the inclusion of some patients without true hypoxemia may have contributed to the high observed median oxygen saturation of 95%. Furthermore, pulse oximetry is not perfectly accurate [33] and we may have included patients with false or brief transient hypoxemia. For example, we included 16 children with documented hypoxemia who survived without receiving respiratory support. These sampling limitations potentially contributed to an underestimation of mortality from hypoxemia. Finally, selection and reporting biases were unavoidable. The site and patient representation varied across RCS globally, and data availability and accuracy were dependent on clinician diagnosis and reporting. Despite these limitations, we believe the study findings are generalizable to pediatric populations in RCS and informative to future work.

## Conclusions

This study demonstrated that children with hypoxemia in RCS are at increased risk of mortality when respiratory care resources are limited. Additionally, despite the new PALICC-2 criteria, PARDS remains challenging to diagnose in RCS, and the presence of a PARDS trigger, as a proxy for PARDS, was not associated with mortality. These findings suggest that mortality may depend more on other factors such as resource availability rather than underlying pathophysiology in RCS. Therefore, ongoing work to strengthen systems and optimize respiratory care resources should be prioritized to reduce inequities. This includes ensuring availability of pulse oximetry to consistently identify children with hypoxemia, oxygen therapy as a minimum basic standard of care, and more advanced infrastructure where feasible. Future initiatives should also focus on global engagement and support of pediatric healthcare providers to promote the implementation of PALICC-2 criteria in RCS to improve diagnosis and management of PARDS. Collectively, these efforts can help clinicians risk-stratify children, allocate available limited resources, and tailor management to optimize clinical outcomes.

## Supplementary Information

Supplementary material 1.

## Data Availability

Study materials, including the template data collection forms, data dictionary, deidentified data used for all analyses, analytic code, and other study-related materials are publicly available at https://borealisdata.ca/dataverse/Pedi/_SepsisCoLab. Approval from the PALISI Global Health sub-group and a signed data access agreement will be required for publications related to these data.

## Abbreviations

BiPAP: Bilevel positive airway pressure
COVID-19: Coronavirus disease of 2019
CPAP: Continuous positive airway pressure
HFNC: High-flow nasal cannula
ICU: Intensive care unit
IQR: Interquartile range
LODS: Lambaréné Organ Dysfunction Score
LOS: Length of stay
LRTI: Lower respiratory tract infection
PALICC-2: Second Pediatric Acute Lung Injury Consensus Conference
PARDS: Pediatric acute respiratory distress syndrome
PARITY: Paediatric Acute Critical Illness Point Prevalence Study
PICU: Pediatric intensive care unit
RCS: Resource-constrained setting(s)
SD: Standard deviation
SDI: Socio-demographic index
STROBE: Strengthening the Reporting of Observational Studies in Epidemiology
WHO: World Health Organization
