# Supplementary material for: Risk factors for mortality in children with hypoxemia in resource-constrained settings: a secondary analysis of Global Paediatric Acute Critical Illness Point Prevalence Study (PARITY)
Source: BMC Glob Public Health. 2026 Jan 9;4:5. doi: 10.1186/s44263-025-00238-7 (PMC12790117; doi:10.1186/s44263-025-00238-7)
Supplement: Supplementary file 1 — Supplementary material 1. [file 44263_2025_238_MOESM1_ESM.docx]

Supplementary Material 1: Table of Contents

Case Report Forms 1

Pediatric Acute Care Infrastructure and Resource Availability 1

Initial Intake Survey 9

Daily Assessment Survey 16

Final Outcomes Survey 22

Supplemental Tables 26

Table S1. STROBE Statement 26

Table S2. Criteria for PARDS, Possible PARDS and At-risk for PARDS according to PALICC-2^1^ 28

Table S3. Resources reported to be “Always” or “Often” available by socio-demographic index (SDI) 28

Table S4. Resource utilization by hypoxemic children with or without a PARDS trigger. 29

Table S5. Data missingness in patients with hypoxemia and a PARDS trigger by highest respiratory support. 30

Table S6. Data missingness in patients with hypoxemia and a PARDS trigger by socio-demographic index. 31

Table S7. Characteristics of hypoxemic children admitted to resource-constrained hospitals by survival status. 31

Table S8. Resource utilization by hypoxemic children admitted to resource-constrained hospitals by survival status. 33

Table S9. Multivariable Logistic Regression Model for the Association between Respiratory Resource Bundle Availability and Mortality. 34

Table S10. Multivariable Logistic Regression Model for the Association between PARDS Trigger and Mortality. 34

Supplemental Figures 35

Fig S1. Map of Global PARITY Participating Sites 35

Fig S2. Directed acyclic graph illustrating the potential effect of respiratory resource availability on mortality and confounders. 36

Fig S3. Directed acyclic graph illustrating the potential effect of PARDS trigger diagnosis on mortality and confounders. 37

Fig S4. Resource availability by SDI (socio-demographic index) quintile. 37

Fig S5. Flowchart displaying PARDS-related diagnoses according to PALICC-2 criteria based on highest respiratory support. 38

Global PARITY Investigator Authors 39

Authors and Affiliations 39

Contributions of Global PARITY Investigator Authors 44

Study Collaborators 45

References 46

# Case Report Forms

## Pediatric Acute Care Infrastructure and Resource Availability

The objective of the following questionnaire is to describe the current infrastructure of pediatric acute care among PARITY and non-PARITY study sites.

1. To describe the resources and staffing of hospital pediatric emergency departments, general wards, intermediate care or high dependency units (HDUs) and intensive care units (ICUs).
2. To describe the availability of basic and advanced acute care resources in the hospital wards. Consent

By responding to this questionnaire, you are indicating your consent to participate in this study. For further questions, please contact:

Fiona Muttalib [(Fiona.muttalib@mail.utoronto.ca)](mailto:(Fiona.muttalib@mail.utoronto.ca)

#### ** Please note: For optimal viewing on a mobile device, display the survey horizontally. **

Please specify your healthcare role Administrator Consultant physician Registered nurse

Resident or registrar physician Medical student

Medical intern

Clinical or medical officer Other

(If multiple responses apply, please select the

role in which you spend the majority of your work hours.)

If other, please specify

Please select your country of practice Country drop-down menu

*Hospital Characteristics*

##### Please provide your PARITY site ID (if applicable) Please provide the name of your hospital

Please provide the complete address of your hospital

##### (e.g. street number, street name, city, town or village as applicable)

Is your hospital funded by the public sector (e.g. government, health ministry), private sector (e.g. charitable organization, non-governmental Both organization) or both?

Is your hospital affiliated to a medical school or schools?

Please provide the name of the medical school(s) and university affiliated to your hospital.

Does your hospital/university have any of the following training programs (select all that apply)

Please indicate which of the following pediatric subspecialties are available in your institution

Private Public Both

Don’t know Yes

No

Nursing (diploma/degree)

Specialty nursing (critical care or emergency care) Clinical officer (undergraduate)

Specialty clinical officer (critical or emergency care)

Doctor (accredited internship site) Specialty pediatric doctor (residency in pediatrics)

Subspecialty acute care pediatrics (fellowship in intensive care, pediatric intensive care or pediatric emergency medicine)

Pediatric Subspecialty drop-down menu

Is your hospital in a rural or urban area? Rural Urban

Unsure/Don't know

Does your hospital have a busy season over the course of the year?

Yes No

(e.g. increased patient volume during rainy season, winter season, etc)

#### Please select the HIGH season months (months during which the number of admitted patients is higher than average) and LOW season months (months during which the number of admitted patients is lower than average) below:

High season Jan Feb Mar April May June July Aug Sept Oct Nov Dec

Low season Jan Feb Mar April May June July Aug Sept Oct Nov Dec

#### Emergency Department or Outpatient Pediatric Department Characteristics. The following questions pertain to whichever department in your hospital where children are seen for emergency visits, referred to here as the "emergency department.

Where are acute pediatric visits seen in your institution? Pediatric emergency department (medical/surgical)

Pediatric emergency department (medical only) Mixed adult and pediatric emergency department Other outpatient clinic with acute presentations

What is the average number of daily pediatric emergency visits to your emergency department?

0-100

101 - 500

501 - 1000

> 1000

Is your emergency department open 7 days per week? Yes No

Is your emergency department open 24 hours per day? Yes No

Does your emergency department use a triage score Or system for every child ?

Please specify the name of the triage system currently in use.

Please upload any documents or photos of triage documents currently in use in your institution Who performs triage of children at arrival to the emergency department or hospital? (Select all that apply)

If other, please specify

Are visual emergency care aids available in your emergency department?

**Pediatric Inpatient Ward Characteristics** What is the average number of pediatric hospital admissions per day in your institution?

What is the average number of pediatric inpatients on any given weekday (Monday to Friday) at 09h00?

Yes No

Registered nurse Medical intern Medical student Clinical officer

Medical resident/registrar Consultant physician Other

Yes No

(e.g. standardized algorithms or protocols, wall charts for selection or dosing of medicines or fluid administration)

Admissions drop-down menu

Census drop-down menu

What is the minimum age of patients admitted to the pediatric ward or general inpatient ward of your hospital?

What is the maximum age of children admitted to your pediatric ward or general inpatient ward?

What is the estimated hospital child mortality per 100 admitted children (%) in your institution?

Select all of the following pediatric wards that exist in your institution

Age drop-down menu (months)

Age drop-down menu (years)

Mortality drop-down menu

General pediatrics ward

Pediatric inpatients on general adult ward

Pediatric high-dependency or intermediate care unit Pediatric step-down unit

Pediatric intensive care unit (ICU) Pediatric beds in adult intensive care unit Pediatric oncology ward

Pediatric surgical ward Pediatric malnutrition ward

Neonatal intensive care unit or ward

(Intermediate care, step-down or high-dependency unit may describe a designated ward for sick children outside the ICU where children may be provided additional monitoring or therapies that are not available on the general ward.)

#### Average number of patients per DOCTOR on the Pediatric Intensive Care Unit or for children admitted to the adult Intensive Care Unit

Note: If a single officer/doctor provides care for patients across several wards during a given period (e.g. nighttime), indicate the total number of children across the wards per officer/doctor.

1-5 6-10 11-1 16-2 21-3 21-4 41-5 51-6 61-7 71-8 81-9 91-1 >100

Daytime (e.g. 07h00 - 19h00) Nighttime (e.g. 19h00 - 07h00)

#### Please indicate the average number of patients per NURSE in the Pediatric Intensive Care Unit or for children in the adult Intensive Care Unit.

##### Daytime (e.g. 07h00 - 19h00) Nighttime (e.g. 19h00 - 07h00)

What is the maximum frequency of routine vital sign measurement and recording in the Pediatric Intensive Care Unit or Adult Intensive Care Unit?

Frequency drop-down menu

#### Please indicate the most senior healthcare provider available in-house on the Pediatric

**Intensive Care Unit or Adult Intensive Care Unit, and on the general pediatric or general inpatient ward**

Weekday daytime (Monday - Friday, 07h00 - 19h00) Weekend daytime (Saturday - Sunday, 07h00 - 19h00) Weekday nighttime (Monday - Friday, 19h00 - 07h00) Weekend nighttime (Saturday - Sunday, 19h00 - 07h00)

Student doctor Registrar or Resident

Medical or Clinical Officer Nurse

General doctor Pediatrician Anesthe-tist Ped-intensivist

#### Please indicate whether the following equipment is available in the Pediatric Intensive Care

**Unit or in the adult Intensive Care Unit where children are admitted and on the General Pediatric ward**

Electricity

Back-up power supply or generator

Running water and soap OR alcohol-based hand rub Weighing scale

Oxygen (piped oxygen delivery system) Oxygen cylinder

Oxygen concentrator

Pediatric self-inflating bag and pediatric masks Pediatric laryngoscopes and endotracheal tubes

Pediatric nasal prongs or simple facemask for low flow oxygen High flow oxygen delivery device

Bubble CPAP

Continuous or bilevel positive airway pressure (CPAP, BiPAP) device Conventional mechanical ventilator

High frequency oscillatory ventilation Nebulizer

Suction device and suction catheters Continuous oximetry monitor Continuous cardiac monitor Invasive arterial pressure

Never Rarely Sometimes Often Always Don't know

monitoring device

Blood pressure measuring device with pediatric cuffs Defibrillator

Resuscitation trolley/cart CPR backboard

Infusion or syringe pumps Central line catheters Pediatric cervical spine collars

External ventricular drain (EVD) Intracranial pressure monitor Pediatric nasogastric tubes

Peritoneal dialysis system (catheters, dialysate) Hemodialysis system

Continuous renal replacement system Thermometer

Glucometer and strips

Extra-corporeal life support (e.g.ECMO)

Personal protective equipment

([PPE] e.g. disposable gloves, gown, facemask, goggles)

#### Please indicate the average number of children per OFFICER/DOCTOR on the High Dependency or Intermediate Care ward

Note: If a single officer/doctor provides care for patients across several wards during a given period, indicate the total number of children across the wards per officer/doctor

1-5 6-10 11-1 16-2 21-3 21-4 41-5 51-6 61-7 71-8 81-9 91-1 >100

Daytime (e.g. 07h00 - 19h00) Nighttime (e.g. 19h00 - 07h00)

#### Please indicate the average number of children per NURSE on the High Dependency or Intermediate Care ward

##### Daytime (e.g. 07h00 - 19h00) Nighttime (e.g. 19h00 - 07h00)

What is the maximum frequency of routine vital sign measurement and recording in the General Pediatric Ward

Frequency drop-down menu

#### Please indicate if the following medications are available to children in your hospital

Oral rehydration salts/solution (ORS) Oral Zinc supplement

Isotonic crystalloid (0.9% NaCl or Lactated Ringers or other) Dextrose 5% water (D5W) containing solutions

High dextrose solution (e.g. Dextrose 25% or 50%) Total parenteral nutrition (TPN)

Whole blood

Packed red blood cells Platelets Immunoglobulin

Other component blood products (Albumin, Cryo, FFP) Antipyretic drugs (paracetamol, acetamophen, ibuprofen) Vasoactive drugs (e.g. epinephrine/adrenaline, norepinephrine, etc)

Intravenous opioids (e.g. morphine, fentanyl, hydromorphone) Intravenous, oral or rectal benzodiazepines (lorazepam, midazolam, diazepam)

Never Rarely Sometimes Often Always Don't know

Propofol Ketamine

Beta-lactam antibiotics (e.g. penicillin, ampicillin) Aminoglycoside (e.g. gentamycin, tobramycin) Cephalosporin (e.g. ceftriaxone, cephalexin, cefuroxime) Macrolides (e.g. azithromycin, clarithromycin) Carbapenem (e.g. ertapenem, meropenem) Fluoroquinolones (e.g. ciprofloxacin)

Extended-spectrum

beta-lactams (e.g. piperacillin-tazobactam) Anti-malarial drugs

Intravenous anti-epileptic drugs (e.g. phenytoin, phenobarbital, levetiracetam, benzodiazepines)

Oral anti-epileptic drugs (e.g. phenytoin, phenobarbital, levetiracetam, benzodiazepines)

Inhaled beta-agonists (salbutamol, albuterol, terbutaline)

Intravenous beta-agonists (salbutamol, albuterol, terbutaline)

Steroids (oral or intravenous) Surfactant

Inhaled nitric oxide

Unfractionated or low-molecular weight heparin Tocilizumab

Remdesivir

#### Please indicate whether the following resources for diagnosis are available to children in your hospital?

Chest X-ray (portable or standing ultrasound) Echocardiogram

Computed tomography Magnetic resonance imaging Electrocardiogram machine Blood gas analysis

Serum lactate Complete blood count Manual differential Thin and thick smear

Malaria rapid diagnostic test (MRDT)

Serum electrolytes and creatinine Urine analysis

Cerebrospinal fluid analysis Cerebrospinal fluid cultures Blood culture

Respiratory syncytial virus PCR Influenza virus PCR

COVID-19 PCR

COVID-19 serology C-reactive protein Ferritin

Troponin

Serum brain natriuretic peptide D-dimer

Fibrinogen

Partial Thromboplastin Time (PTT) International Normalized Ratio (INR) Erythrocyte sedimentation rate Albumin

Never Rarely Sometimes Often Always Don't know

#### Please indicate whether the following allied health service professionals are available for pediatric consultations in your hospital.

Pharmacist Dietician

Respiratory Therapist Physiotherapist Occupational therapist Social services Psychologist

Never Rarely Sometimes Often Always Don't know

#### Institutional Ethics Review and Approval Processes

What is the frequency of FULL ethics committee review in your institution?

What date was your application for institutional ethics review SUBMITTED to your institution? (DD-MM-YY)

What date was your application for institutional

ethics review APPROVED by your institution? (DD-MM-YY)

Did your institutional ethics review committee (or equivalent) require FULL BOARD review of the Global PARITY study prior to granting approval?

Were there costs associated with institutional ethics review?

What was the total cost of institutional ethics review in United States Dollars (USD)

Did your institutional ethics review committee approve WAIVED CONSENT by the patient?

Did your institutional ethics review committee require a Data Sharing Agreement

Please provide the date of data sharing agreement submission (DD-MM-YY)

Please provide the date of data sharing agreement approval (DD-MM-YY).

Is translation of study protocols from English into your local language mandatory for ethics review?

Is a professional translation certificate required?

Does your hospital allow for ethics approval from a separate institution, without requiring local ethics review?

Can this separate institution be outside of your country?

Does your institutional ethics review committee require legal review of protocol or data sharing agreements?

Are there non-clinical research staff employed in your hospital?

Weekly

Every 1 - 2 months

Every 3 - 6 months

> 6 months

Yes No

Yes No

Yes No

(In the case of waived consent, informed written consent is NOT required from participating patients)

**Yes No**

**Yes No**

## Initial Intake Survey

record ID field

#### Study Tracking

Enrollment Period 1

2

3

4

5—July 2022

Enter Site ID (Example: SA26) Your Initials

#### Demographic and Anthropometric Data

Biologic Sex Female

Male

not reported

(what is the patient's assigned sex at birth)

Patient Age Under 2 years

Over 2 years

If patient under 2 years of age, write age in months If patient over 2 year of age, write age in years Weight (kg)

Height or Length (cm)

Mid-Upper Arm Circumference (MUAC) (cm)

#### Presentation/Admission Data

Was this patient directly admitted or hospitalized bypassing your hospital's emergency department?

Yes No

What is the admission source? Operating room Transfer or referral Outpatient source

Admission Location Inpatient Ward

High-Dependency Unit (HDU) Intermediate Care Unit (IMCU) Intensive Care Unit (ICU) Other

If other, please describe

#### Initial Vital Signs Available/Recorded (Yes or No)

##### Refers to the first set of available vital signs on presentation/admission. All vital signs/measurements should be from the same assessment

Vital Signs Available/Recorded at (or closest to) 0800

Heart Rate No/Yes

Respiratory Rate No/Yes

Blood Pressure No/Yes

Oxygen Saturation No/Yes

Temperature No/Yes

AVPU Score (measured or calculable) No/Yes

Glasgow Coma Scale No/Yes

Blantyre Coma Scare No/Yes

Heart Rate

Respiratory Rate in breaths/minute Systolic Blood Pressure in mmHg Diastolic Blood Pressure in mmHg Oxygen Saturation

Was this saturation obtained while the patient was receiving any source of oxygen?

Temperature in degrees C AVPU Scale

(Alert-Verbal-Pain-Unresponsive)

Glasgow Coma Scale: Total Score

Yes No

Not documented

Alert Verbal Pain

Unresponsive

Glasgow Coma Scale: EYE 1: Does not open eyes

2: Open eyes in response to pain 3: Open eyes in response to voice 4: Open eyes spontaneously

Glasgow Coma Scale: VERBAL 1: Makes no sound 2: Makes sound

3: Words

4: Confused, disoriented speech 5: Oriented speech

Glasgow Coma Scale: MOTOR 1: Makes no movement

2: Abnormal extension to painful stimuli 3: Abnormal flexion to painful stimuli 4: Withdrawal from painful stimuli

5: Localizes to painful stimuli 6: Obeys Commands

Blantyre Coma Scale: Total Score

Was the mental status score (AVPU, GCS, BCS) calculated while the patient was on continuous sedation for more than 4 hours?

Yes No

No documented

#### Signs and Symptoms

##### Select yes if any of the following are listed in the medical record as a symptom or sign in the history of present illness or review of systems at the time of admission

Vomiting Everything Yes

No

No documented

Inability to feed Yes

No

No documented

Seizure or Convulsion (observed or reported) Yes No

No documented

#### Physical Exam Findings

##### For all physical exam findings, select yes if the finding is listed in the medical record as a physical finding observed at the time of admission

Sunken Eyes Yes

No

No documented

Slow skin pinch Yes

No

No documented

Severe Pallor Yes

No

No documented

Jaundice Yes

No

No documented

Prostration Yes

No

No documented

Coma Yes

No

No documented

Deep Breathing Yes

No

No documented

If there is deep breathing, please describe. Select all that apply

Capillary Refill Time (in seconds)

Rapid, shallow breathing Nasal flaring

Grunting

Chest in-drawing Accessory muscle use Obstructed breathing Wheezing

Stridor Crepitations Central cyanosis Cough

Pulse Quality Normal

Bounding Thready

Not documented

#### Outcomes and Disposition

Disposition upon Discharge from Emergency Department Discharged home

Operating Room/Operating Theater Admitted to Inpatient Service Transferred to Other Facility

Death

Absconded or left against medical advice

If transferred, was the patient transferred for a higher level of care?

Yes No

No documented

Location upon admission to inpatient service Inpatient Ward

High-Dependency Unit (HDU) Intermediate Care Unit (IMCU) Intensive Care Unit (ICU) Other

Not documented

Please give details if admitted to other inpatient Setting

Length of Emergency Department Stay (in hours)

Location upon leaving operating room/operating theater Inpatient Ward

High-Dependency Unit (HDU) Intermediate Care Unit (IMCU) Intensive Care Unit (ICU) Death

Pediatric Overall Performance Categrory Normal

Mild Disability Moderate Disability Severe Disability

Coma or vegetative state Brain death

Not able to determine

Is disability due to physical or mental injury? No

Disability due to physical injury Disability due to mental injury

Disability due to both physical and mental injury Unknown

Not documented

#### Final Emergency Department Diagnoses, Admission Diagnoses, or Underlying Causes of Death

##### Select the most appropriate diagnosis or underlying cause of death from the list that best matches the physician's diagnoses (presumptive, likely, suspected, or final)

What is the primary ED or admission diagnosis, or underlying cause of death?

Communicable and nutritional conditions Non-communicable diseases

Injuries

Ill-defined or cause unknown

Choose the most appropriate diagnosis Pneumonia Bronchiolitis

Upper respiratory tract infection or croup Tuberculosis

Diarrhea/gastroenteritis Hepatitis

Measles Pertussis Tetanus

Urinary tract infection or pyelonephritis Acute otitis media

Pharyngitis

HIV/AIDS or AIDS-related illness Sepsis or septic shock

Acute Malaria

Multisystem Inflammatory Syndrome in Children (MISC)

Acute COVID-19

Any skin or soft tissue infection Malnutrition

Meningitis or Encephalitis Fever and neutropenia

Other infectious or parasitic disease

Please indicate specific forms of malnutrition (select all that apply)

List other type of malnutrition

If OTHER infectious or parasitic disease, please describe

Wasting

all that apply) Failure to thrive Kwashiorkor

Severe acute malnutrition (SAM) Stunting

Marasmus Other

Not documented

Choose the most appropriate diagnosis Congenital malformations Birth Asphyxia Prematurity

Hydrocephalus (with or without a VPS) Stroke

Status Epilepticus or seizure Heart Failure

Diabetes or related complication (diabetic ketoacidosis, hyperglycemia, hypoglycemia) Bowel obstruction

Intussusception Appendicitis

Gastrointestinal bleed (upper or lower) Peptic ulcer disease/GERD/Reflux Constipation

Pancreatitis Cancer/malignancy Allergies, allergic rhinitis Asthma/Status Asthmaticus

Chronic Respiratory or lung Disease Sickle cell disease/anemia or associated complication (acute chest, pain crisis) Hypovolemia/Dehydration

Shock

Anemia

Renal failure or injury Carbon monoxide poisoning

Other non-communicable diseases

What is the cancer or oncologic diagnosis?

Indicate whether acute or chronic kidney or renal injury

Acute Chronic

Not documented

Indicate the type of shock

Cardiogenic Neurogenic Anaphylactic Hemorrhagic

Hypovolemic due to dehydration Obstructive

Other

Not documented

Other type of shock

Indicate the type of stroke

Non-traumatic hemorrhagic stroke Ischemic

Other

Not documented

List or describe other kind of stroke

If OTHER non-communicable disease, please describe

Choose the most appropriate diagnosis Traumatic brain injury Polytrauma

Fracture Laceration

Non-accidental trauma or child abuse Self-injury or suicide attempt

Assault Fall Drowning

Poisoning/Ingestion Burn

Envonmation by either bite or sting Foreign body aspiration

Foreign body ingestion Other injury

If OTHER injury, please describe If ill-defined, please describe

Would you like to add a SECONDARY diagnosis Yes No

Would you like to add a TERTIARY diagonsis Yes No

#### Co-Morbid Conditions

For all comorbid conditions, select the most appropriate conditions that best match conditions listed in the problem list or past medical history

Asthma/reactive airways disease Yes

No/No documented history

Congenital Heart Disease Yes

No/No documented history

Select specific congenital heart lesion, if known. Choose all that apply.

Suspected but unable to confirm Ventricular septal defect (VSD) Atrial septal defect (ASD) Tetralogy of Fallot (TOF) Patent ductus arteriosus (PDA) Truncus arteriosus (TA)

Transposition of the great arteries (TGA) Total or partial anomalous venous drainage Undifferentiated "cyanotic" heart disease Undifferentiated "acyanotic" heart disease Other

If "other" type of congenital heart disease, note it here

Rheumatic Heart Disease Yes

No/No documented history

Human Immunodeficiency Virus (HIV) Negative Positive Exposed

Unknown/Not documented

Malnutrition Yes

No/No documented history

Indicate malnutrition type Wasting

Failure to thrive Kwashiorkor

Severe acute malnutrition (SAM) Stunting

Marasmus Other

Not documented

Indicate other type of malnutrition

Cancer/malignancy Yes

No/No documented history

What is the cancer diagnosis

Obesity Yes

No/No documented history

Diabetes Yes

No/No documented history

Development Delay Yes

No/No documented history

Cerebral Palsy Yes

No/No documented history

Seizure disorder or epilepsy or fits Yes

No/No documented history

Hydrocephalus Yes

No/No documented history

Sickle cell disease/anemia Yes

No/No documented history

Thalassemia Yes

No/No documented history

Hypertension Yes

No/No documented history

Genetic or congenital condition Yes

No/No documented history

Please describe any genetic or congenital conditions

Any other co-morbid condition not listed above? Yes /No

Any other co-morbid condition? Add one per field

Would you like to add another comorbid condition?

Yes/No

Any other co-morbid condition? Add one per field

Would you like to add another comorbid condition?

Yes/No

Any other co-morbid condition? Add one per field

Would you like to add another comorbid condition? Yes/No Any other co-morbid condition?

Add one per field

Any additional comments

## Daily Assessment Survey

record ID field

###### Study tracking

Your Initials

This is the REGIONAL code (XX), followed by your SITE NUMBER (12), followed by the PATIENT NUMBER (000) XX-12-000

| Hospital Day | 0 (day of presentation/admission) |
| --- | --- |
|  | 1 |
|  | 2 |
|  | 3 |
|  | 4 |
|  | 5 |
|  | 6 |
|  | 7 |

###### Patient Location at the beginning and end of the day

Patient location at the START (0800) of the DAY

Patient location at the END (2000) of the DAY

Emergency Department


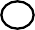


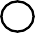


Inpatient Ward


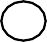


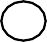


High- Dependenc y Unit (HDU)


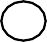


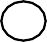


Intermediate Care

Unit (IMCU)


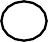


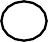


Intensive Care Unit (ICU)


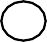


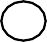


Operating room (OR)


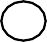


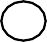


Morgue (dead)


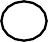


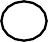


Discharged/ transferr ed/ AMA


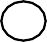


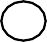


Other


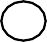


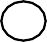


Were any laboratory or imaging studies obtained on this day?

Yes No

#### Laboratory and Diagnostic Data

Select "yes" if a value is recorded in the medical record; "no" if not measured or recorded. First measurement after presentation to the emergency department, on admission to the hospital if direct admit, or first value of the day if the patient is admitted. All CBC (Hgb, platelets) and chemistries (Cr, BUN) measurements should be from the same lab draw.

White blood cell count (WBC) Yes/No

Absolute lymphocyte percent (ALC) Yes/No

Hemoglobin (HGB) Yes/No

Platelet count Yes/No

Glucose Yes/No

Blood urea nitrogen (BUN) Yes/No

AST/SGOT Yes/No

ALT/SGPT Yes/No

Bilirubin Yes/No

Lactate dehydrogenase (LDH) Yes/No

| Creatinine Serum pH | Yes/No Yes/No |  |
| --- | --- | --- |
| Lactate  Partial thromboplastin time (PTT) INR | Yes/No Yes/No Yes/No |  |
| D-dimer Fibrinogen | Yes/No Yes/No |  |
| Ferritin  Erythrocyte sedimentation rate (ESR) | Yes/No Yes/No |  |
| C-reactive protein (CRP)  Brain natriuretic peptide (BNP) Head imaging | Yes/No Yes/No Yes/No |  |
| Chest imaging Abdominal imaging Echocardiogram | Yes/No Yes/No Yes/No |  |
| Total white blood cell count (WBC) (in 10^9) |  |  |
| Absolute LYMPHOCYTE count (%) |  |  |
| Serum hemoglobin (g/DL) |  |  |
| Hemoglobin units (mmol/L) |  |  |

Platelet Count (in 10^9)

Glucose

Add "0" for undetectably LOW Add "800" of undetectably HIGH

Glucose (mg/dL; mmol/L)

Blood urea nitrogen (mg/dL; mmol/L) Serum AST/SGOT in IU (in IU/L) Serum ALT/SGPT in IU (in IU/L) Serum Bilirubin (Total)

Bilirubin Units (mg/dL; SI) Lactate dehydrogenase (LDH)

Lactate dehydrogenase (units/L; SI units) Creatinine (mg/dL; micromol/L)

Serum pH

pH blood source Arterial

Venous Capillary Unknown

Serum Lactate

Lactate blood source Arterial

Venous Capillary Unknown

Lactate blood units (mg/dL; mmol/L (SI units))

Partial Thromboplastin Time (PTT) (in seconds) INR

D-dimer (NANOgram/mL; MICROgram/mL) Fibrinogen (mg/dL; g/L (SI unit))

Erythrocyte sedimentation rate in mm/hr

C reactive protein (MILIgrams/L (SI units); MICROgrams/mL Brain natriuretic peptide (BNP)

in NANOgrams/L

Head Imaging Computed tomography (CT)

Magnetic resonance imaging (MRI) Head ultrasound

Head imaging results

Chest Imaging Computed tomography (CT)

Magnetic resonance imaging (MRI) Ultrasound

Plain x-ray

Please indicate which quadrants on chest imaging contain opacifications. Choose all that apply

Chest imaging results

Right Upper Lobe Middle/Lower Lobe Left Upper Lobe Left Lower Lobe None

Abdominal Imaging Computed tomography (CT)

Magnetic resonance imaging (MRI) Ultrasound

Plain x-ray

Abdominal imaging results Echocardiogram Results

###### Did the patient receive any of the following therapies/interventions?

Select "yes" for all of the following therapies administered during that hospital day (time of presentation/0000 to 2359)

Continuous Sedation >4 hrs Yes/No

Invasive Mechanical Ventilation Yes/No

Non-Invasive Postive Pressure Yes/No

Simple or Low-Flow Oxygen Therapy Yes/No

Fluid Bolus Yes/No

Blood or Blood Product Transfusion Yes/No

Vasoactives Yes/No

Corticosteroids Yes/No

Antibiotic therapy Yes/No

Anti-malarial therapy Yes/No

Anti-viral therapy Yes/No

Anti-fungal therapy Yes/No

Anticoagulation (low-molecular weight heparin, heparin infusion)

Yes/No

Dialysis (any type) Yes/No

Procedure (bedside or surgical) Yes/No

Chest Compressions Yes/No

Does this patient have a suspected or proven diagnosis of COVID-10 or MIS-C? Yes

No

If acute COVID-19 or MIS-C associated with COVID-19, which of the following therapies were used?

Corticosteroid Remdesivir Tociluzimab Monoclonal antibodies IVIG

Anticoagulation (low-molecular weight heparin, heparin infusion)

Aspirin

None of the above Other

List other therapy administered for COVID or MIS-C

Select which corticosteroid (select all the apply) Hydrocortisone Dexamethasone Methylprednisolone Prednisone

Other

List other steroid

Select the type of anti-coagulation Prophylactic

Therapeutic

Not documented

Which medications were used for continuous sedation? Fentanyl

List the other medications if other was selected

Remifentanyl or sufentanyl Morphine

Hydromorphone Midazolam Alprazolam Lorazepam Dexmedetomidine Propofol Ketamine

Inhaled volatile anaesthetics Other

How long did the patient receive continuous sedation? < 6hrs 6-12hrs

12-18hrs

>18hrs

Not documented

If the patient received vasoactives, please indicate the type. Epinephrine (Adrenalin)

the type. Norepinephrine (Noradrenalin) Dopamine

Dobutamine Vasopressin Phenylephrine Milrinone Other

If patient received other vasoactives, please list

What type of dialysis (select all that apply) Intermittent hemodialysis (HD)

Continuous renal replacement therapy (CRRT, CVVH, CVVHD)

Peritoneal dialysis Not documented

What procedure(s) did the patient receive (select all that apply)? Central line placement including HD catheter

PICC line placement Arterial line placement Lumbar puncture

Chest tube/pigtail placement/Thoracentesis Paracentesis/Peritoneal drain placement Extraventricular drain (EVD)/Intracranial pressure monitor (bedside)

Bronchoscopy Incision and drainage

Interventional or diagnostic procedure (cardiac cath, interventional radiology, EGD, colonoscopy) Surgical procedure in the operating room

Other

List interventional procedure here: List surgical procedure here:

List other procedure here:

#### pARDS Questions

What was the highest level of respiratory support Invasive mechanical ventilation

received by the patient?

What is the oxygen saturation 6 hours after starting

Invasive mechanical ventilation CPAP or Bipap

High Flow Oxygen

Simple or Low Flow Oxygen None (was in room air)

mechanical ventilation? (as a %)

What is the fraction of inspired oxygen 6 hours after

starting mechanical ventilation? (as a %)

What is the mean airway pressure 6 hours after

starting mechanical ventilation? (in cmH20)

If the patient received mechanical ventilation, please indicate the type

If other mode of invasive mechanical ventilation, please describe

Conventional

High-frequency oscillatory ventilation JET

Other

If the patient received non-invasive positive pressure CPAP

ventilation, indicate the type. Bubble CPAP

BiPap

High-flow nasal cannula Other

If other type of non-invasive positive pressure, please describe

What type of interface is used to provide non-invasive positive pressure ventilation?

If the patient received simple or low-flow oxygen, indicate the type

If other type of low-flow oxygen was used, please describe

Nasal cannula/prongs Nasal-only pillows

Oro-nasal mask (any mask that covers both nose and mouth)

Not documented Nasal cannula

Face mask of any type (simple, non-rebreather, reservoir)

Other

Not documented

###### Vital Signs Available/Recorded at (or closest to) 0800

Heart Rate No/Yes

Respiratory Rate No/Yes

Blood Pressure No/Yes

Oxygen Saturation No/Yes

Temperature No/Yes

AVPU Score (measured or calculable) No/Yes

Glasgow Coma Scale No/Yes

Blantyre Coma Scare No/Yes

Heart Rate

Respiratory Rate in breaths/minute Systolic Blood Pressure in mmHg Diastolic Blood Pressure in mmHg Oxygen Saturation

Was this saturation obtained while the patient was receiving any source of oxygen?

Temperature in degrees C

Yes No

Not documented

AVPU Score Alert

Voice Pain

Unresponsive

Glasgow Coma Scale: Total Score

Glasgow Coma Scale: EYE 1: Does not open eyes

2: Open eyes in response to pain 3: Open eyes in response to voice 4: Open eyes spontaneously

Glasgow Coma Scale: VERBAL 1: Makes no sound 2: Makes sound

3: Words

4: Confused, disoriented speech 5: Oriented speech

Glasgow Coma Scale: MOTOR 1: Makes no movement

2: Abnormal extension to painful stimuli 3: Abnormal flexion to painful stimuli 4: Withdrawal from painful stimuli

5: Localizes to painful stimuli 6: Obeys Commands

Blantyre Coma Scale: Total Score

Was the mental status score (AVPU, GCS, BCS) calculated while the patient was on continuous sedation for more than 4 hours?

Number of Stools (if counted)

Urine output in ml/kg (if calculated)

Fluid balance for the 24 hour period (or since admission if Day 0)

Use [-] for negative fluid balance Use [+] for positive fluid balance Or enter Not Recorded

Any additional comments

## Final Outcomes Survey

record ID field leave blank

#### Study Tracking

Your Initials Enter Patient ID

This is the REGIONAL code (XX), followed by your

SITE NUMBER (12), followed by the PATIENT NUMBER (000) XX-12-000

#### Outcome

Hospital Outcome Discharged home

Transferred to other facility Death

Absconded or left against medical advice Alive and still admitted on Day 31

If transferred, was the patient transferred for a higher level of care?

Yes No

Not documented

On which Hospital Day did the final outcome occur? 0 (day of presentation/admission)

1

2

3

4

5

6

7

Day 8-30

> 30 days

Not Documented

On what hospital day did the outcome occur?

#### Final Diagnoses or Underlying Causes of Death

##### For patients alive but still admitted on Day 30, select the current working diagnoses

What is the primary discharge diagnosis or underlying cause of death?

Communicable and nutritional conditions Non-communicable diseases

Injuries

Ill-defined or cause unknown

Choose the most appropriate diagnosis Pneumonia Bronchiolitis

Upper respiratory tract infection or croup

Tuberculosis Diarrhea/gastroenteritis Hepatitis

Measles Pertussis Tetanus

Urinary tract infection or pyelonephritis Acute otitis media

Pharyngitis

HIV/AIDS or AIDS-related illness Sepsis or septic shock

Acute Malaria

Multisystem Inflammatory Syndrome in Children (MISC)

Acute COVID-19

Any skin or soft tissue infection Malnutrition

Meningitis or Encephalitis Fever and neutropenia

Other infectious or parasitic disease

Please indicate specific forms of malnutrition (select all that apply)

List other type of malnutrition

Please give details for other infectious cause or parasitic disease

Wasting

Failure to thrive Kwashiorkor

Severe acute malnutrition (SAM) Stunting

Marasmus Other

Not documented

Choose the most appropriate diagnosis Congenital malformations Birth Asphyxia Prematurity

Hydrocephalus (with or without a VPS) Stroke

Status Epilepticus or seizure Heart Failure

Diabetes or related complication (diabetic ketoacidosis, hyperglycemia, hypoglycemia) Bowel obstruction

Intussusception Appendicitis

Gastrointestinal bleed (upper or lower) Peptic ulcer disease/GERD/Reflux Constipation

Pancreatitis Cancer/malignancy Allergies, allergic rhinitis Asthma/Status Asthmaticus

Chronic Respiratory or lung Disease Sickle cell disease/anemia or associated complication (acute chest, pain crisis) Hypovolemia/Dehydration

Shock Anemia

Renal failure or injury Carbon monoxide poisoning

Other non-communicable diseases

What is the cancer or oncologic diagnosis?

Indicate whether acute or chronic kidney or renal disease

Acute Chronic

Not documented

If shock, please indicate type Cardiogenic

Neurogenic

List other type of shock

Anaphylactic Hemorrhagic

Hypovolemic due to dehydration Obstructive

Other

Not documented

Indicate the type of stroke Non-traumatic hemorrhagic stroke Ischemic

Other

Not documented

List or describe other kind of stroke

If other non-communicable disease, please describe

Choose the most appropriate diagnosis Traumatic brain injury Polytrauma

Fracture Laceration

Non-accidental trauma or child abuse Self-injury or suicide attempt

Assault Fall Drowning

Poisoning/Ingestion Burn

Envonmation by either bite or sting Foreign body aspiration

Foreign body ingestion Other injury

If other injury, please describe

Please describe ill-defined or undefined cause

Would you like to add a SECONDARY discharge diagnosis or underlying cause of death?

Would you like to add a TERTIARY discharge diagnosis or underlying cause of death?

Yes No

Yes No

#### Pathogen identification

Was a definitive pathogen(s) identified during hospitalization?

Yes No

Not documented

How many pathogens were definitively identified? 1

2

3

4

Pathogen 1 name:

Pathogen 1 source (select all the apply): Blood

Cerebrospinal fluid (CSF) Sputum

Urine

Nasopharyx or oropharynx Abscess

Other

If other source of pathogen, please describe Pathogen 2 name:

Pathogen 2 source (select all the apply): Blood

Cerebrospinal fluid (CSF) Sputum

Urine

Nasopharyx or oropharynx Abscess

Other

If other source of pathogen, please describe Pathogen 3 name:

Pathogen 3 source (select all the apply): Blood

Cerebrospinal fluid (CSF) Sputum

Urine

Nasopharyx or oropharynx Abscess

Other

If other source of pathogen, please describe Pathogen 4 name:

Pathogen 4 source (select all the apply): Blood

Cerebrospinal fluid (CSF) Sputum

Urine

Nasopharyx or oropharynx Abscess

Other

If other source of pathogen, please describe

#### Pediatric Overall Performance Category

Pediatric Overall Performance Category Normal

Mild Disability Moderate Disability Severe Disability

Coma or vegetative state Brain death

Not able to determine

Is disability due to physical or mental injury? No

Disability due to physical injury Disability due to mental injury

Disability due to both physical and mental injury Unknown

Not documented

Any additional comments

# Supplemental Tables

## Table S1. STROBE Statement

Checklist of items that should be included in reports of ***cohort studies***

#### Item

**No. Recommendation**

#### Page No.

###### Title and abstract

|  | | (*b*) Provide in the abstract an informative and balanced summary of what was done and  what was found | Abstract, pages 3-4 |
| --- | --- | --- | --- |
| Introduction |  |  |  |
| Background/ | 2 | Explain the scientific background and rationale for the investigation being reported | Background, page |
| rationale |  |  | 4 |
| Objectives | 3 | State specific objectives, including any prespecified hypotheses | Background, last |
|  |  |  | sentence, page 6 |
| Methods |  |  |  |
| Study design | 4 | Present key elements of study design early in the paper | Methods, page 4 |
|  |  |  |  |
| Setting | 5 | Describe the setting, locations, and relevant dates, including periods of recruitment, | Global PARITY |
|  |  | exposure, follow-up, and data collection | dataset and Participating sites section of |
|  |  |  | Methods, pages 6-8 |
|  |  |  |  |
| Participants | 6 | (*a*) Give the eligibility criteria, and the sources and methods of selection of participants. | Patient population section of Methods, |
|  |  | Describe methods of follow-up | Pages 9-12 |
|  |  |  |  |
|  |  | (*b*) For matched studies, give matching criteria and number of exposed and unexposed | N/A |
| Variables | 7 | Clearly define all outcomes, exposures, predictors, potential confounders, and effect | Respiratory care |
|  |  | modifiers. Give diagnostic criteria, if applicable | resources and Patient population sections of Methods, pages 8-12 |
|  |  |  |  |
| Data sources/ | 8* | For each variable of interest, give sources of data and details of methods of assessment | Respiratory care |
| measurement |  | (measurement). Describe comparability of assessment methods if there is more than | resources and |
|  |  | one group | Patient population sections of Methods, pages 8-12 |
| Bias | 9 | Describe any efforts to address potential sources of bias | Second to last paragraph of Conclusions, pages 25-26 |
| Study size | 10 | Explain how the study size was arrived at | Patient population section of Methods, |
|  |  |  | pages 9-12 |
| Quantitative | 11 | Explain how quantitative variables were handled in the analyses. If applicable, describe | Statistical |
| variables |  | which groupings were chosen and why | analysis section |
|  |  |  | of the Methods, |
|  |  |  | pages 13-14 |
| Statistical | 12 | (*a*) Describe all statistical methods, including those used to control for confounding | Statistical analysis |
| methods |  |  | section, pages 13-14 |
|  |  | (*b*) Describe any methods used to examine subgroups and interactions |  |
|  |  |  |  |
|  |  | (*c*) Explain how missing data were addressed |  |
|  |  |  |  |
|  |  | (*d*) If applicable, explain how loss to follow-up was addressed |  |
|  |  |  |  |
|  |  | (*e*) Describe any sensitivity analyses |  |
|  |  |  |  |
| Results |  |  |  |
| Participants | 13* | (a) Report numbers of individuals at each stage of study—eg numbers potentially | Patient |
|  |  | eligible, examined for eligibility, confirmed eligible, included in the study, completing | characteristics |
|  |  | follow-up, and analysed | section of Results, pages 15-18 |
|  |  | (b) Give reasons for non-participation at each stage | Figure 1 |

1 (*a*) Indicate the study’s design with a commonly used term in the title or the abstract Title, page 1

|  |  | (c) Consider use of a flow diagram | Figure 1 |
| --- | --- | --- | --- |
| Descriptive data | 14* | (a) Give characteristics of study participants (eg demographic, clinical, social) and | Patient |
|  |  | information on exposures and potential confounders | characteristics, |
|  |  |  | section of Results, pages 15-18. See also |
|  |  |  | Table 1 |
|  |  | (b) Indicate number of participants with missing data for each variable of interest | Table 1 |
|  |  | (c) Summarise follow-up time (eg, average and total amount) | Outcomes section of Results, last paragraph, page 20 |
| Outcome data | 15* | Report numbers of outcome events or summary measures over time | Outcomes section of Results, pages 18-20 |
| Main results | 16 | (*a*) Give unadjusted estimates and, if applicable, confounder-adjusted estimates and | Outcomes section of |
|  |  | their precision (eg, 95% confidence interval). Make clear which confounders were | Results, pages 18-20 |
|  |  | adjusted for and why they were included |  |
|  |  | (*b*) Report category boundaries when continuous variables were categorized | Outcomes section of Results, pages 18-20 |
|  |  | (*c*) If relevant, consider translating estimates of relative risk into absolute risk for a | N/A |
|  |  | meaningful time period |  |
| Other analyses | 17 | Report other analyses done—eg analyses of subgroups and interactions, and sensitivity | Diagnosing PARDS section of Results, |
|  |  | analyses | Pages 20-21 |
|  |  |  |  |
| Discussion |  |  |  |
| Key results | 18 | Summarise key results with reference to study objectives | Conclusions, pages 22-27 |
| Limitations | 19 | Discuss limitations of the study, taking into account sources of potential bias or | Second to last |
|  |  | imprecision. Discuss both direction and magnitude of any potential bias | paragraph of Conclusions, pages 25-26 |
| Interpretation | 20 | Give a cautious overall interpretation of results considering objectives, limitations, | Final paragraph of |
|  |  | multiplicity of analyses, results from similar studies, and other relevant evidence | Conclusions, pages 26-27 |
| Generalisability | 21 | Discuss the generalisability (external validity) of the study results | Second to last paragraph of Conclusions, page 26 |
| Other information |  |  |  |
| Funding | 22 | Give the source of funding and the role of the funders for the present study and, if | N/A |
|  |  | applicable, for the original study on which the present article is based |  |

##### *Give information separately for exposed and unexposed groups.

**Note:** An Explanation and Elaboration article discusses each checklist item and gives methodological background and published examples of transparent reporting. The STROBE checklist is best used in conjunction with this article (freely available on the Web sites of PLoS Medicine at [http://www.plosmedicine.org/,](http://www.plosmedicine.org/) Annals of Internal Medicine at [http://www.annals.org/,](http://www.annals.org/) and Epidemiology at [http://www.epidem.com/).](http://www.epidem.com/)) Information on the STROBE Initiative is available at [http://www.strobe-statement.org.](http://www.strobe-statement.org/)

## Table S2. Criteria for PARDS, Possible PARDS and At-risk for PARDS according to PALICC-2^1^

|  | **PARDS** | **Possible PARDS** | **At-risk for PARDS** |
| --- | --- | --- | --- |
| *Exclude perinatal lung disease* | Yes | Yes | Yes |
| *Known PARDS trigger within 7 days* | Yes | Yes | Yes |
| *Edema not fully explained by cardiac failure or fluid overload* | Yes | Yes | Yes |
| *Chest imaging with new opacities (unilateral or bilateral)* | Yes | - Yes, if nasal interface  - No, if IMV/NIV | Yes |
| *Respiratory interface/support* | - ETT  - NIV full facemask (PEEP ≥ 5 cm H20) | - NIV nasal interface or HFNC (≥ 1.5L/kg/min or ≥ 30L/min) with CXR  - ETT or NIV full facemask without CXR available | Any interface including simple mask/nasal cannula |
| *Oxygenation criteria* | IMV: OSI ≥ 5  NIV: SpO2/FiO2 ≤ 250 | SpO2/FiO2 ≤ 250 | Oxygen supplementation to maintain SpO2 ≥ 88% (FiO2 > 21% via IMV/NIV or meeting flow threshold via simple mask or nasal cannula) |

PARDS=pediatric acute respiratory distress syndrome, ETT=endotracheal tube, NIV=non-invasive ventilation, PEEP=positive end expiratory pressure, IMV=invasive mechanical ventilation, OSI=oxygen saturation index, SpO2=oxygen saturation, FiO2=fraction of inspired oxygen, CXR=chest x-ray

## Table S3. Resources reported to be “Always” or “Often” available by socio-demographic index (SDI)

| **Resource** | **All sites, (n=46)** | **Low SDI, (n=10)** | **Low-middle SDI, (n=13)** | **Middle SDI, (n=13)** | **High-middle SDI, (n=10)** |
| --- | --- | --- | --- | --- | --- |
| **Basic Bundle, n (%)** | **43 (93)** | **8 (80)** | **13 (100)** | **12 (92)** | **10 (100)** |
| Oxygen, n (%) | 43 (93) | 10 (100) | 10 (77) | 13 (100) | 10 (100) |
| Nasal prongs/simple mask, n (%) | 45 (98) | 10 (100) | 13 (100) | 12 (92) | 10 (100) |
| Self-inflating bag/mask, n (%) | 43 (93) | 8 (80) | 13 (100) | 12 (92) | 10 (100) |
| Suction, n (%) | 43 (93) | 9 (90) | 12 (92) | 12 (92) | 10 (100) |
| Nebulizer, n (%) | 45 (98) | 9 (90) | 13 (100) | 13 (100) | 10 (100) |
| Pulse oximetry, n (%) | 45 (98) | 9 (90) | 13 (100) | 13 (100) | 10 (100) |
| **Intermediate Bundle, n (%)** | **32 (70)** | **5 (50)** | **7 (54)** | **10 (77)** | **10 (100)** |
| Intubation supplies, n (%) | 38 (83) | 8 (80) | 8 (62) | 12 (92) | 10 (100) |
| Conventional mechanical ventilation, n (%) | 33 (72) | 6 (60) | 8 (62) | 10 (77) | 9 (90) |
| High-flow nasal cannula, n (%) | 35 (76) | 7 (70) | 6 (46) | 12 (92) | 10 (100) |
| Non-invasive positive pressure, n (%) | 33 (72) | 6 (60) | 7 (54) | 10 (77) | 10 (100) |
| Chest x-ray, n (%) | 44 (96) | 9 (90) | 12 (92) | 13 (100) | 10 (100) |
| Steroids, n (%) | 45 (98) | 10 (100) | 13 (100) | 12 (92) | 10 (100) |
| Sedation, n (%) | 44 (96) | 9 (90) | 13 (100) | 12 (92) | 10 (100) |
| Vasoactives, n (%) | 45 (98) | 9 (90) | 13 (100) | 13 (100) | 10 (100) |
| **Advanced Bundle, n (%)** | **19 (41)** | **1 (10)** | **1 (8)** | **9 (69)** | **8 (80)** |
| High frequency oscillatory ventilation, n (%) | 20 (43) | 1 (10) | 2 (15) | 9 (69) | 8 (80) |
| Inhaled nitric oxide, n (%) | 17 (37) | 1 (10) | 1 (8) | 9 (69) | 6 (60) |
| Respiratory therapist, n (%) | 28 (61) | 3 (30) | 6 (46) | 12 (92) | 7 (70) |
| Blood gas analysis, n (%) | 34 (74) | 5 (50) | 6 (46) | 13 (100) | 10 (100) |
| Computerized tomography, n (%) | 37 (80) | 8 (80) | 7 (54) | 12 (92) | 10 (100) |
| **Expert Bundle, n (%)** | **3 (7)** | **0 (0)** | **0 (0)** | **0 (0)** | **3 (30)** |
| Extracorporeal membrane oxygenation, n (%) | 3 (7) | 0 (0) | 0 (0) | 0 (0) | 3 (30) |

## Table S4. Resource utilization by hypoxemic children with or without a PARDS trigger.

| **Resource utilization** | **Total**  **(N=763)** | **PARDS trigger**  **(N=430)** | **No PARDS trigger**  **(N=333)** | **p-value** |
| --- | --- | --- | --- | --- |
| Highest level of respiratory support required |  | | | 0.53 |
| Invasive mechanical ventilation, n (%) | 53 (6.9) | 27 (6.3) | 26 (7.8) |  |
| Non-invasive positive pressure, n (%) | 42 (5.5) | 27 (6.3) | 15 (4.5) |  |
| High-flow nasal cannula, n (%) | 99 (13) | 53 (12) | 46 (14) |  |
| Simple oxygen, n (%) | 553 (72) | 316 (73) | 237 (71) |  |
| No support, n (%) | 16 (2.1) | 7 (1.6) | 9 (2.7) |  |
| Other resources |  | | | |
| Chest X-ray, n (%) | 286 (37) | 207 (48) | 79 (24) | **<0.001** |
| Continuous sedation >4hrs, n (%) | 55 (7.2) | 30 (7.0) | 25 (7.5) | 0.78 |
| Vasoactive, n (%) | 30 (3.9) | 14 (3.3) | 16 (4.8) | 0.28 |

P-values < 0.05 represent a significant difference in resource distribution between groups, calculated with a chi-square test.

## Table S5. Data missingness in patients with hypoxemia and a PARDS trigger by highest respiratory support.

| **Data missingness** | **IMV (n=27)** | **NIV (n=27)** | **HFNC (n=53)** | **Simple O2 (n=316)** |
| --- | --- | --- | --- | --- |
| CXR not done, n(%) | 9 (33) | 13 (48) | 21 (40) | 173 (55) |
| FiO2 not reported six hours after IMV/NIV, n(%) | 8 (30) | 19 (70) | 53 (100) | - |
| SpO2 not reported six hours after IMV/NIV, n(%) | 3 (11) | 14 (52) | 53 (100) | 316 (100) |
| MAP not reported six hours after IMV/NIV, n(%) | 11 (41) | 27 (100) | - | - |
| NIV interface not reported, n(%) | - | 7 (26) | - | - |
| O2 liter flow not reported, n(%) | - | - | 53 (100) | 316 (100) |
| Insufficient data for PARDS diagnosis, n(%) | 15 (56)* | 27 (100)^ | - | - |
| Insufficient data for Possible PARDS diagnosis, n(%) | 11 (41)** | 23 (85)^^ | 53 (100) | - |
| Insufficient data for At-risk for PARDS diagnosis, n(%) | 13 (48)*** | 20 (74)^^^ | 53 (100) | 316 (100) |

IMV=invasive mechanical ventilation, NIV=non-invasive ventilation, HFNC=high-flow nasal cannula, O2=oxygen, CXR=chest x-ray, FiO2=fraction inspired oxygen, SpO2=oxygen saturation, MAP=mean airway pressure, PARDS=pediatric acute respiratory distress syndrome; *CXR done and FiO2, SpO2, MAP reported six hours after IMV; **Sufficient data for PARDS diagnosis or FiO2, SpO2, MAP reported six hours after IMV and no CXR done; ***CXR done and FiO2 reported six hours after IMV; ^CXR done and FiO2, SpO2, MAP reported six hours after NIV; ^^CXR done, NIV nasal interface reported and SpO2, FiO2 reported six hours after NIV; ^^^CXR done and FiO2 reported six hours after NIV.

## Table S6. Data missingness in patients with hypoxemia and a PARDS trigger by socio-demographic index.

| **Data missingness** | Low SDI  (n=202) | Low-middle SDI (n=83) | Middle SDI  (n=91) | High-middle SDI (n=54) |
| --- | --- | --- | --- | --- |
| CXR not done, n(%) | 137 (68) | 50 (60) | 17 (19) | 19 (35) |
| FiO2 missing*, n(%) | 16/22 (73) | 6/14 (43) | 2/9 (22) | 3/9 (33) |
| SpO2 missing*, n(%) | 10/22 (45) | 5/14 (36) | 1/9 (11) | 1/9 (11) |
| MAP missing*, n(%) | 20/22 (91) | 9/14 (64) | 3/9 (33) | 6/9 (67) |
| NIV interface missing**, n(%) | 7/15 (47) | 0/9 (0) | 0/0 (N/A) | 0/3 (0) |

PARDS=pediatric acute respiratory distress syndrome; SDI=socio-demographic index, CXR=chest x-ray, FiO2=fraction inspired oxygen, SpO2=oxygen saturation, MAP=mean airway pressure, IMV=invasive mechanical ventilation, NIV=non-invasive ventilation. *If highest level of respiratory support was IMV or NIV: IMV/NIV was the highest level of support for 22 patients in Low SDI countries, 14 patients in Low-middle SDI countries, 9 patients in Middle SDI countries, and 9 patients in High-middle SDI countries. **If highest level of respiratory support was NIV: NIV was the highest level of support for 15 patients in Low SDI countries, 9 patients in Low-middle SDI countries, 0 patients in Middle SDI countries, and 3 patients in High-middle SDI countries.

## Table S7. Characteristics of hypoxemic children admitted to resource-constrained hospitals by survival status.

| **Characteristics** | **Total**  **(N=763)** | **Survivors**  **(N=711)** | **Non Survivors**  **(N=52)** | **Missing data**  **N(%)** | **p-value** |
| --- | --- | --- | --- | --- | --- |
| Age (years), median(IQR) | 1.9 (0.6-5) | 1.9 (0.5-4) | 3.5 (0.7-7.5) | 0 (0) | **0.02** |
| Female, n(%) | 336 (44) | 309 (40) | 27 (52) | 1 (0.1) | 0.48 |
| Weight (kilograms), median (IQR) | 10 (6.5-16) | 10 (6.5-16) | 10.5 (5.7-20) | 77(10) | 0.75 |
| SpO2 on admission, median (IQR) | 95 (91-98) | 95 (91-98) | 94 (83-98) | 35(4.7) | 0.19 |
| Malnutrition, n (%) | 197 (26) | 176 (25) | 21 (40) | **-** | **0.01** |
| PARDS triggers |  | | | | |
| Any PARDS trigger, n(%) | 430 (56) | 404 (57) | 26 (50) | - | 0.34 |
| Lower respiratory tract infection*, n(%) | 366 (48) | 353 (50) | 13 (25) | **-** | **0.001** |
| Pneumonia, n(%) | 256 (34) | 244 (34) | 12 (23) | - | 0.1 |
| Bronchiolitis, n(%) | 116 (15) | 116 (16) | 0 (0) | **-** | **0.002** |
| COVID-19, n(%) | 17 (2.2) | 16 (2.3) | 1 (1.9) | - | 0.88 |
| Sepsis, n(%) | 58 (7.6) | 48 (6.8) | 10 (19) | **-** | **0.001** |
| Trauma, n(%) | 20 (2.6) | 17 (2.4) | 3 (5.8) | - | 0.14 |
| Other, n(%) | 6 (0.8) | 3 (0.4) | 3 (5.8) | **-** | **<0.001** |
| Global Burden of Disease Region |  | | | **-** | **<0.001** |
| SSA, n (%) | 267 (35) | 234 (33) | 33 (63) |  |  |
| SA, n (%) | 222 (29) | 207 (29) | 15 (29) |  |  |
| LA, n (%) | 169 (22) | 167 (23) | 2 (3.8) |  |  |
| CE, n (%) | 22 (2.9) | 21 (3.0) | 1 (1.9) |  |  |
| NA, n (%) | 15 (2.0) | 15 (2.1) | 0 (0) |  |  |
| SLA, n (%) | 68 (8.9) | 67 (9.4) | 1 (1.9) |  |  |
| Socio-demographic Index |  | | | **-** | **<0.001** |
| Low, n(%) | 343 (45) | 314 (44) | 29 (56) |  |  |
| Low-Middle, n(%) | 168 (22) | 148 (21) | 20 (38) |  |  |
| Middle, n(%) | 168 (22) | 166 (23) | 2 (3.8) |  |  |
| High-Middle, n(%) | 84 (11) | 83 (12) | 1 (1.9) |  |  |
| **Respiratory resource bundle availability** |  | | | - | **<0.001** |
| No bundle | 81 (11) | 71 (10) | 10 (19) |  |  |
| Basic | 273 (36) | 254 (36) | 19 (37) |  |  |
| Intermediate | 177 (23) | 156 (22) | 21 (40) |  |  |
| Advanced | 189 (25) | 188 (26) | 1 (1.9) |  |  |
| Expert | 43 (5.6) | 42 (5.9) | 1 (1.9) |  |  |

P-values < 0.05 represent a significant difference in characteristic distribution between groups, calculated with a chi-square test (categorical variables) or Wilcoxon rank-sum test (continuous variables). *Lower respiratory tract infection includes patients with pneumonia, bronchiolitis, and/or COVID-19. IQR=interquartile range; SpO2=oxygen saturation; PARDS=pediatric acute respiratory distress syndrome; SSA=Sub-Saharan Africa; SA=South Asia; LA=Latin America and Caribbean; CE=Central Europe, Eastern Europe, and Central Asia; NA=North Africa and Middle East; SLA=Southern Latin America.

## Table S8. Resource utilization by hypoxemic children admitted to resource-constrained hospitals by survival status.

| **Resource utilization** | **Total**  **(N=763)** | **Survivors**  **(N=711)** | **Non Survivors**  **(N=52)** | **Missing data**  **N(%)** | **p-value** |
| --- | --- | --- | --- | --- | --- |
| Highest level of respiratory support required |  | | | - | **<0.001** |
| Invasive mechanical ventilation, n (%) | 53 (6.9) | 48 (6.8) | 5 (9.6) |  |  |
| Non-invasive positive pressure, n (%) | 42 (5.5) | 33 (4.6) | 9 (17) |  |  |
| High-flow nasal cannula, n (%) | 92 (12) | 84 (12) | 15 (29) |  |  |
| Simple oxygen, n (%) | 553 (72) | 530 (75) | 23 (44) |  |  |
| No support, n (%) | 16 (2.1) | 16 (2.3) | 0 (0) |  |  |
| Other resources |  | | | | |
| Chest x-ray, n (%) | 286 (37) | 276 (39) | 10 (19) | **-** | **0.005** |
| Continuous sedation >4hrs, n (%) | 55 (7.2) | 44 (6.2) | 11 (21) | - | **<0.001** |
| Vasoactive medications, n (%) | 30 (3.9) | 18 (2.5) | 12 (23) | - | **<0.001** |

P-values < 0.05 represent a significant difference in resource distribution between groups, calculated with a chi-square test.

## Table S9. Multivariable Logistic Regression Model for the Association between Respiratory Resource Bundle Availability and Mortality.

| **Variable** | **Reference Category** | **Adjusted OR (95% CI)** | **p-value** |
| --- | --- | --- | --- |
| Respiratory Resource Bundle Available | Expert or Advanced | 18 (4.1 - 83) | <0.001 |
| Average Pediatric Inpatient Census | Greater than 50 patients | 0.54 (0.29 - 0.98) | 0.04 |
| PICU Availability | Yes | 1.6 (0.90 - 3.0) | 0.11 |
| Academic Center Designation | Yes | 1.5 (0.57 - 4.2) | 0.39 |

OR: Odds ratio; CI: Confidence interval; PICU: Pediatric Intensive Care Unit

## Table S10. Multivariable Logistic Regression Model for the Association between PARDS Trigger and Mortality.

| **Variable** | **Reference Category** | **Adjusted OR (95% CI)** | **p-value** |
| --- | --- | --- | --- |
| PARDS Trigger | Yes | 0.56 (0.30 – 1.0) | 0.07 |
| Respiratory Resource Bundle Available | Expert or Advanced | 13 (3.0 - 54) | 0.001 |
| Age | Six years or older | 3.1 (1.6 – 5.8) | 0.001 |
| Malnutrition | Yes | 2.0 (1.1 - 3.6) | 0.03 |
| LODS | Greater than 0 | 3.7 (1.8 – 7.6) | <0.001 |

OR: Odds ratio; CI: Confidence interval; PARDS: Pediatric Acute Respiratory Distress Syndrome; LODS: Lambaréné Organ Dysfunction Score

# Supplemental Figures


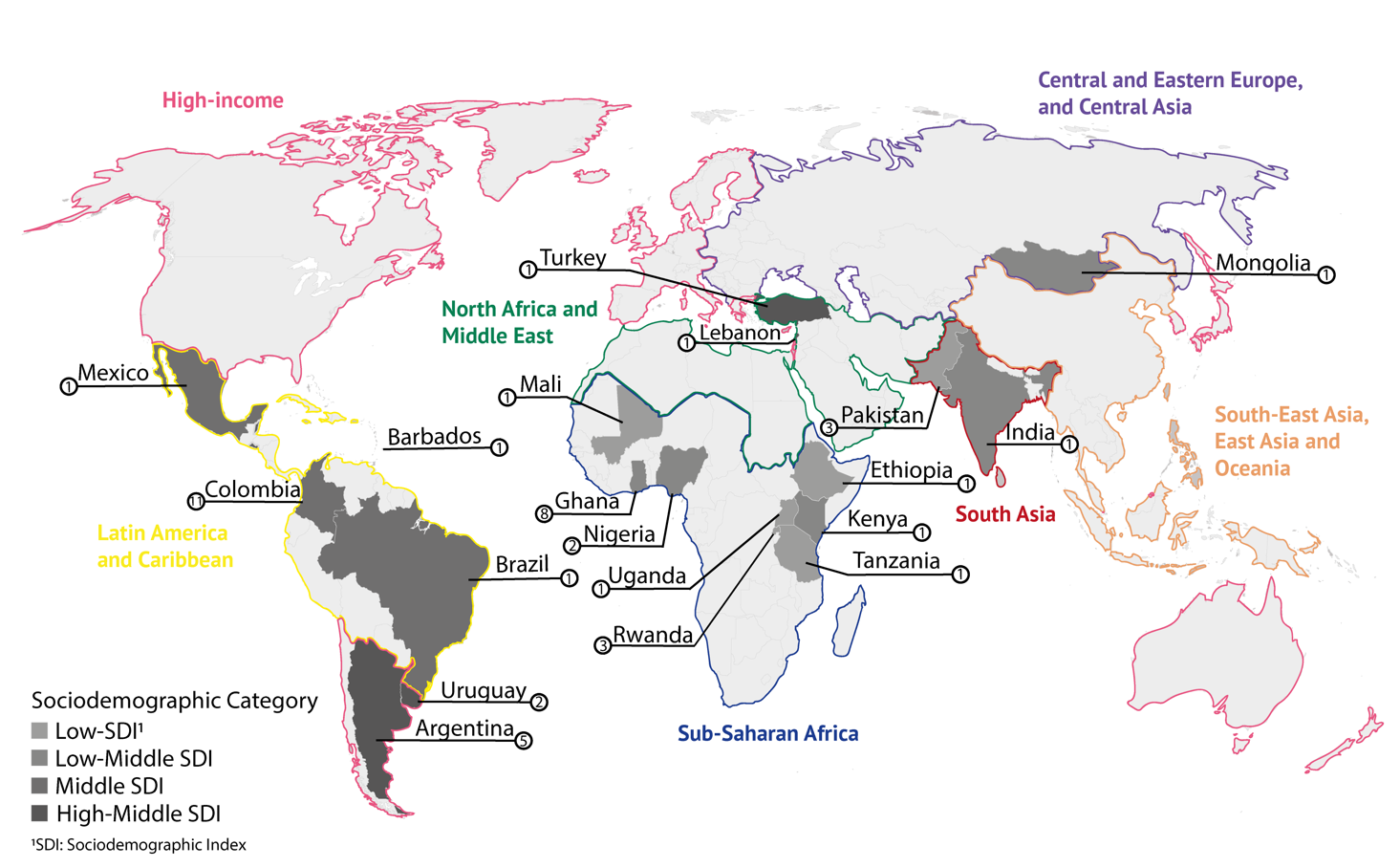


##

## Fig. S1. Map of Global PARITY Participating Sites

The Global Burden of Disease Super Regions are outlined and labeled in color. Participating site sociodemographic category (low-, low-middle-, middle-, and high-middle socio-demographic index [SDI] quartile) is shown in greyscale.


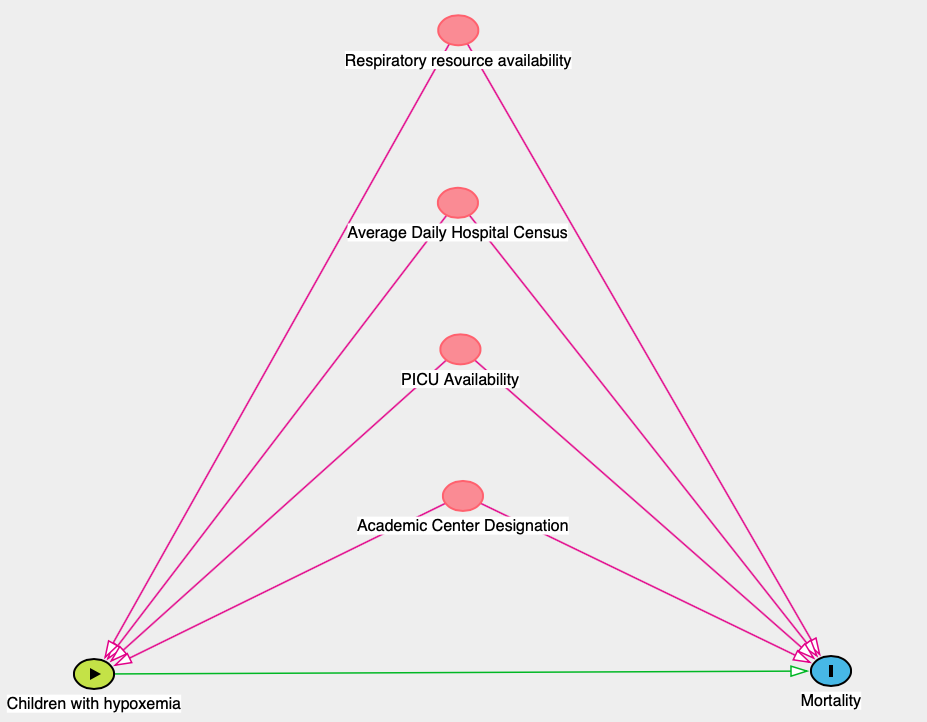


## Fig. S2. Directed acyclic graph illustrating the potential effect of respiratory resource availability on mortality and confounders


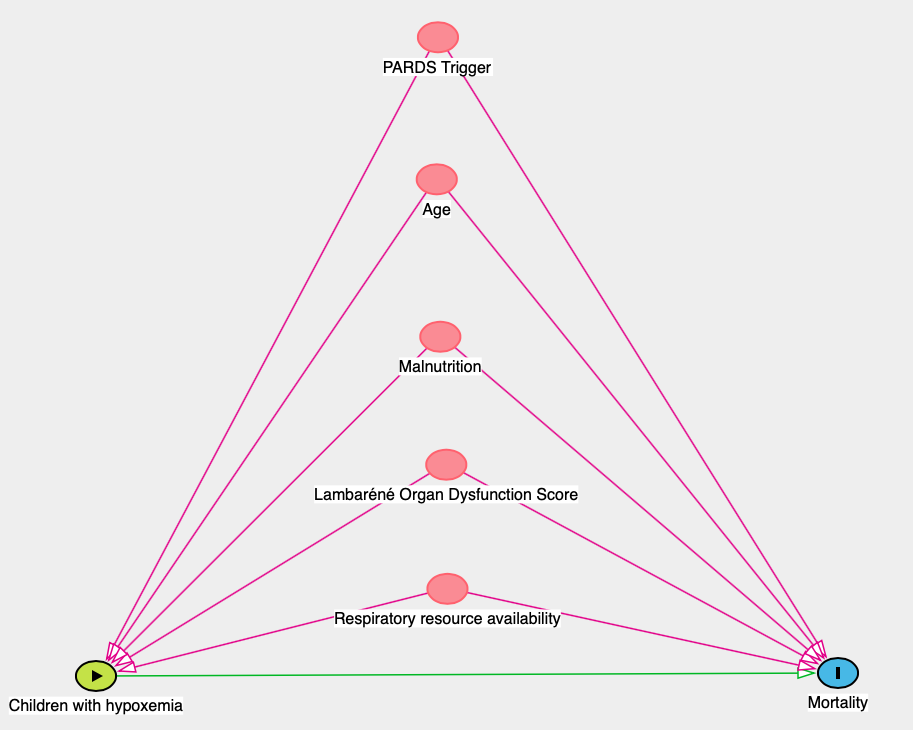


## Fig. S3. Directed acyclic graph illustrating the potential effect of PARDS trigger diagnosis on mortality and confounders


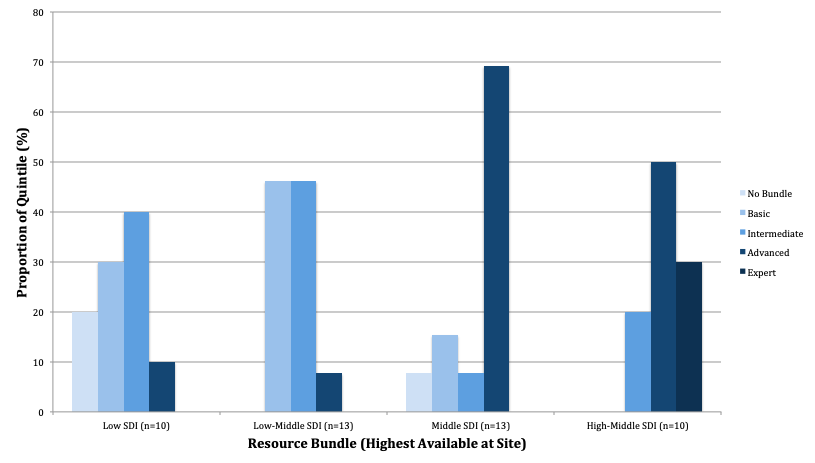


## Fig. S4. Resource availability by SDI (socio-demographic index) quintile


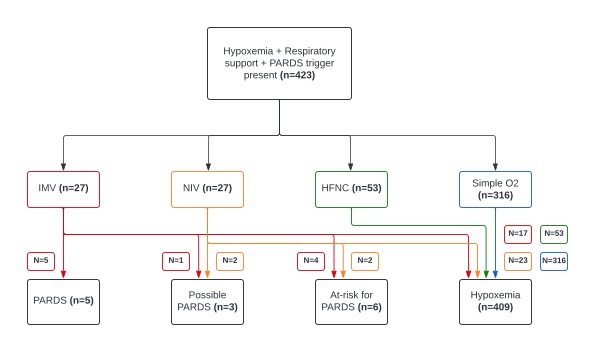


## Fig. S5. Flowchart displaying PARDS-related diagnoses according to PALICC-2 criteria based on highest respiratory support

## PARDS = pediatric acute respiratory distress syndrome, IMV = invasive mechanical ventilation, NIV = non-invasive ventilation, HFNC = high-flow nasal cannula, O2 = oxygen.

# Global PARITY Investigator Authors

## Authors and Affiliations

This is a list of authors who comprise the Global PARITY Investigators group. Authors and their Affiliations are listed alphabetically by last name.

**Alhassan Abdul-Mumin, MD**

Department of Paediatrics and Child Health, University for Development Studies, School of Medicine, and Tamale Teaching Hospital, Tamale, Ghana

**Nabisere Allen, BS**

Department of Jinja Regional, Referral Hospital, Jinja, Uganda

**Paloma Amarillo, MD**

Departamento de Emergencia Pediátrica, Hospital Pereira Rossell, Montevideo, Uruguay

**Kokou Hefoume Amegan-Aho**

Department of Paediatrics and Child Health, University of Health and Allied Sciences, Ho Volta Region, Ghana

**John Appiah, MD**

Pediatric Intensive Care Unit, Komfo Anokye Teaching Hospital, Kumasi, Ghana

**Pamela Celeste Arancibia, PhD**

Pediatric Critical Care Unit, Hospital Público Materno Ínfantil, Salta, Argentina

**Anita Arias, MD**

Division of Critical Care and Pulmonary Medicine, Department of Pediatrics, St. Jude Children’s Research Hospital, Memphis, Tennessee, USA

**Fehmina Arif, FCPS**

Department of Paediatrics, Dow Medical College/Dow University of Health Sciences, Dr. Ruth Pfau Civil Hospital Karachi, Karachi, Pakistan

**Liliana Yanneth Arteaga, MD**

Departamento de Pediatría, Clínica Imbanaco Grupo Quirón Salud, Cali, Colombia

**Jacqueline Gyapomaa Asibey, MD**

Department of Paediatrics and Child Health, Holy Family Hospital, Techiman Holy Family Hospital Techiman, Ghana

**Jonah Attebery, MD**

Department of Pediatrics, Section of Pediatric Critical Care, University of Colorado, Aurora, Colorado, USA, and Barrow Global Health, Barrow Neurological Institute, Phoenix, Arizona, USA

**Nataly Ávila Guerrero, MD**

Department of Pediatrics, Clínica el Rosario Sede el Tesoro, Medellín, Antioquia, Colombia

**Tigist Bacha, MD**

Department of Pediatrics and Child Health, St. Paul's Hospital Millennium Medical Collage, Addis Ababa, Ethiopia

**Briam Damian Beltran Hernandez, MPH**

Subred Integrada de Servicios Salud Norte, Simon Bolivar, Bogotá, Colombia

**Hippolyte Bwiza Muhire, MD**

Department of Pediatrics, Rwamagana Provincial Hospital, Rwamagana, Rwanda

**Professor Juan Sebastián Calderon-Cardenas, MD**

Pediatric Department – Internation, Hospital Simon Bolivar, Subred Integrada De Servicios De Salud Norte, and Fundacion Cardioinfantil, La Cardio, Hospital Simon Bolivar, Universidad El Bosque, Fundación Universitaria Sanitas, Bogotá, Colombia

**Professor Jhon Camacho-Cruz, MD**

Department of Pediatrics, Universidad Nacional de Colombia, Fundación Universitaria de Ciencias de la Salud, Sociedad de Cirugía de Bogotá-Hospital San José, Fundación Universitaria Sanitas, and Clínica Pediátrica Clínicas Colsanitas, Bogotá, Colombia

**Mariana Lucía Cañete, MD**

Servicio de Emergencias, HIAEP Sor María Ludovica, La Plata, Buenos Aires, Argentina

**Paula Caporal, MD**

Department of International Health, Johns Hopkins Bloomberg School of Public Health, Baltimore, Maryland, USA, and Red Colaborativa Pediátrica de Latinoamérica (LARed Network), La Plata, Buenos Aires, Argentina

**Dulamragchaa Chimedbazar, MPH**

Department of Quality Assurance, National Center for Maternal and Child Health, Ulaanbaatar, Mongolia

**Claudia Patricia Curi, MD**

Santisima Trinidad Children's Hospital, Córdoba City, Córdoba, Argentina

**Karla Emilia de Sa Rodrigues, PhD**

Pediatric Department, Barretos Cancer Hospital, Barretos, São Paulo, Brazil

**Tenywa Emmanuel, MMED**

Paediatrics and Child Health, Jinja Regional Referral Hospital, Jinja, Uganda

**Maria Luisa Escobar, MD**

Department of Pediatric Critical Care Medicine, Goryeb Children's Hospital, Morristown, New Jersey, USA

**Professor Sofia Esposto, MD**

Department of Infectious Diseases, Sor Maria Ludovica Children`s Hospital, La Plata, Buenos Aires, Argentina

**Professor Arieth Figueroa Vargas, MD**

Department of Pediatrics, Clinica Imbanaco Grupo Quiron Salud, Cali, Valle del Cauca, Colombia

**Ericka Fink, MD**

Division of Pediatric Critical Care Medicine, Department of Critical Care Medicine, UPMC Children’s Hospital of Pittsburgh, Pittsburgh, Pennsylvania, USA

**Ana Laura Fustiñana, MD**

Emergency Department, Hospital Garrahan, Ciudad Autónoma de Buenos Aires, Argentina

**Marina Giulietti, MD**

Department of Pediatrics, Hospital Interzonal General de Agudos San Roque de Gonnet, La Plata, Buenos Aires, Argentin a

**Stephanie Gordon Rivera, MPH**

Institute for Global Health Sciences, University of California San Francisco, San Francisco, California, USA

**Muhammad Irfan Habib, FCPS**

Clinical Affairs, ChildLife Foundation, Karachi, Pakistan

**Pascal Havugarurema, MMED**

Department of Pediatrics, Centre Hospitalier Universitaire de Butare, Huye, Rwanda

**David He, MS**

Analytical Solutions Group, North Potomac, Maryland, USA

**Professor Lucia Carolina Hernandez Somerson, MD**

Department of Pediatrics, Hospital de Engativá Bogotá D.C, Colombia

**Nayibe Hincapie Saldarriaga, MD**

Hospitalización Pediátrica, Hospital General de Medellín, Antioquia, Colombia

**Shubhada Hooli, MD**

Department of Pediatrics, Division of Emergency Medicine, Baylor College of Medicine Houston, and Department of Emergency Center, Texas Children's Hospital, Texas, USA

**Jacob Isabirye, MD**

Department of Information Technology, Jinja Regional Referral Hospital, Jinja, Uganda

**Professor Saifullah Jamro, FCPS**

Department of Pediatric, Shaheed Mohtarma Benazir Bhutto Medical University, Larkana Sind, Pakistan

**Professor Juan Camilo Jaramillo-Bustamante, MD**

Department of Pediatric Critical Care, Hospital General de Medellín Luz Castro de Gutiérrez, Department of Pediatric Critical Care, Hospital Pablo Tobón Uribe, Department of Pediatrics, School of Medicine, Universidad de Antioquia, Medellín, Antioquia, Colombia, and Red Colaborativa Pediátrica de Latinoamérica (LARed Network)

**Professor Liliana Patricia Jurado Salcedo, MD**

Departamento de Pediatría, Fundación Universitaria San Martín, Bogotá, Cundinamarca, Colombia

**Halima Kabir, MBBS**

Department of Paediatrics, Bayero University, Aminu Kano Teaching Hospital Kano, Kano, Nigeria

**Caleb Kinyanjui Karanja, Higher National Diploma**

AIC Kijabe Hospital, Kiambu, Kenya

**Adama Mamby Keita, MD**

Center for Vaccines Development-Mali, CVD-MALI, Bamako, Mali, Mali

**Marie-Charlyne Fatima Kilba, MBChB**

Department of Child Health, Greater Accra Regional Hospital, Accra, Ghana

**Niranjan Kissoon, MBBS**

Department of Pediatrics, University of British Colombia, and Institute for Global Health, Children’s and Women’s Hospital, Vancouver, British Columbia, Canada

**Guillermo Kohn-Loncarica, MD**

Departament de Emergencias, Hospital Prof. Dr. Juan P. Garrahan, Buenos Aires, Argentina

**Kandamaran Krishnamurthy, MD**

Department of Pediatric Intensive Care Unit, Queen Elizabeth Hospital, University of West Indies Bridgetown, Barbados

**Jorhk Deiby Lasso Noguera, MD**

Departamento de Pediatría, Hospital Infantil Los Angeles, Pasto, Nariño, Colombia

**Marianne N. Majdalani, MD**

Department of Pediatrics and Adolescent Medicine, American University of Beirut Medical Center, Beirut, Lebanon

**Isabel Cristina Monje Cardona, MD**

Departamento de Pediatría, Hospital de Suba, Bogotá, Cundinamarca, Colombia

**Emilse Noris Montero Nuñez, MD**

Terapia Intensiva Pediátrica, Hospital Público Materno Infantil Salta, Salta, Argentina

**Professor Celia Isabel Mulgado Aguas, MD**

Unidad de Terapia Intensiva Pediátrica, Hospital General León, Universidad de Guanajuato, and Universidad de la Salle Bajío, León, Guanajuato, México

**Raya Yusuph Mussa, MD**

Emergency Department, Muhimbili National Hospital, Dar es Salaam, Tanzania

**Fiona Muttalib, MDCM**

Department of Pediatrics, University of British Colombia, Vancouver, British Columbia, Canada

**John Henry Nebaza, B.Sc.**

Children's Ward, Jinja Regional Referral Hospital, Jinja, Uganda

**Katie Nielsen, MD**

Division of Pediatric Critical Care, Department of Pediatrics, University of Washington, and Department of Global Health, University of Washington, Seattle, Washington, USA

**María Noelia Noya, MD**

Pediatric Emergency Department, Centro Hospitalario Pereira Rossell, Montevideo, Uruguay

**Edna Okaikor Obodai, MD**

Department of Child Health, Cape Coast Teaching Hospital, Cape Coast, Central Region, Ghana

**Carmen Elisa Ocampo, MD**

Research Center, Clínica Imbanaco, Department of Pediatrics, Universidad del Valle, Cali, Valle del Cauca, Colombia

**Çağlar Ödek, MD**

Department of Pediatrics, Bursa Uludağ University, Bursa, Türkiye

**Tagbo Oguonu, MBBS**

Department of Pediatrics, University of Nigeria Teaching Hospital, Ituku/Ozalla, Enugu, Nigeria

**Afua Kwakyewaa Osew-Gyamfi, MD**

Paediatric Unit, Eastern Regional Hospital, Koforidua, Koforidua, Ghana

**Sheila Agyeiwaa Owusu, MD**

Department of Pediatrics and Child Health, University for Development Studies, and Teaching Hospital, Tamale, Ghana, and Clinical Research Department, Faculty of Infectious and Tropical Diseases, London School of Hygiene and Tropical Medicine, London, United Kingdom

**Larko Domeryo Owusu, FGCP**

Directorate of Child Health, Komfo Anokye Teaching Hospital Kumasi, Ashanti Region, Ghana

**Professor Mayerly Milena Palencia Bocarejo, MD**

Departamento de Pediátria, Subrednorte Simon Bolivar- Suba- Engativa Hospital, Bogotá, Colombia

**Professor Freddy Israel Pantoja Chamorro, MD**

Departamento de Pediatría, Hospital Infantil Los Angeles, Pasto, Nariño, Colombia

**Aurora Leonor Pedroza, MD**

Unidad de Terapia Intensiva Pediátrica, Hospital Público Materno Infantil Salta, Salta, Argentina

**Professor Walugembe Simon Peter, MBBS**

Department of Paediatrics, Jinja Regional Referral, Jinja Uganda

**Professor Javier Prego, MD**

Pediatric Emergency Department, Pereira Rossell Hospital Center, Montevideo, Uruguay

**Amal C. Rahi, MPH**

Department of Pediatrics and Adolescent Medicine, American University of Beirut Medical Center, Beirut, Lebanon

**Carmen Rossy Ramírez Hernández, MD**

Department de Pediatría, Hospital Maria Inmaculada, Florencia, Caquetá, Colombia

**Kenneth Remy, MD**

Division of Pulmonary and Critical Care Medicine, Department of Internal Medicine, University Hospitals of Cleveland and Rainbow Babies and Children’s Hospital, and Case Western Reserve University School of Medicine, Cleveland, Ohio, USA

**Pedro Rino, MD**

Área de Emergencias, Hospital de Pediatría Prof. Dr. Juan P. Garrahan, and Universidad de Buenos Aires, Sociedad Latinoamericana de Emergencia Pediátrica (SLEPE), Buenos Aires, Argentina and Red de Investigación y Desarrollo de la Emergencia Pediátrica Latinoamericana (RIDEPLA)

**Adriana Teixeira Rodrigues, PhD**

Departamento de Pediatria, Hospital das Clínicas da Universidade Federal de Minas Gerais, Belo Horizonte, Brazil

**Firas Sakaan, MD**

Department of Global Pediatric Medicine, St. Jude Children’s Research Hospital, Memphis, Tennessee, USA

**Jhuma Sankar, MD**

Department of Pediatrics, All India Institute of Medical Sciences, New Delhi, India

**Professor Hendry Robert Sawe, PhD**

Department of Emergency Medicine, Muhimbili University of Health and Allied Sciences, Dar es Salaam, Tanzania

**Jesus Alberto Serra, MD**

Departamento of Pediatría, Casa de Galicia Montevideo, Montevideo, Uruguay

**Agustin Guido Shaieb, MD**

Terapia Intermedia, Hospital Interzonal de Agudos Sor Maria Ludovica de La Plata, La Plata, Buenos Aires, Argentina

**Arianna McLain Shirk, MD**

Department of Pediatric Emergency Medicine, University of Alabama Birmingham, Birmingham, Alabama, USA

**Enkhtur Shonkhuuz, PhD**

Department of Pediatrics, Children's Hospital National Center for Maternal and Child Health, and National Center for Maternal, Newborn and Women's Health, Ulaanbaatar, Mongolia

**Professor Javier Mauricio Sierra-Abaunza, MSc**

Department of Pediatrics, Hospital General de Medellín, Medellín, Antioquia, Colombia

**Khurram Soomro, FCPS**

Department of Pediatrics, Shaheed Muhtatma Benazir Bhutto Medical University, Larkana Sindh, Pakistan

**Samba Ousmane Sow, MD**

Center for Vaccine Development -Mali (CVD MALI), University of Maryland, Baltimore (UMB), Bamako, Mali

**Abner Vesuvius Tagoola, MD**

Department of Pediatrics, Jinja Regional Referral Hospital, Jinja, Uganda

**Atnafu Mekonnen Tekleab, MD**

Department of Pediatrics and Child Health, St. Paul's Hospital Millennium Medical College, Addis Ababa, Ethiopia

**Margarita Maria Torres, MD**

Pediatric Intensive Care Unit, Clinica Imbanaco Cali, Valle del Cauca, Colombia

**Pablo Vasquez-Hoyos, MSc**

Department of Pediatrics, Universidad Nacional de Colombia, and Departamento de Pedaitría, Sociedad de Cirugía de Bogotá Hospital de San Jose, FUCS, Bogotá, Colombia and Red Colaborativa Pediátrica de Latinoamérica

**Amelie von Saint Andre-von Arnim, MD**

Division of Pediatric Critical Care, Department of Pediatrics, University of Washington, and Department of Global Health, University of Washington, Seattle, Washington, USA

**Justin Qi Yuee Wang, MBBChir**

Paediatric Intensive Care Unit, Royal Brompton Hospital, London, UK

**Rafiuk Cosmos Yakubu, MBChB**

Department of Paediatrics and Child Health, Tamale Teaching Hospital and School of Medicine, University for Development Studies, Tamale, Northern Region, Ghana

**Rita Fosu Yeboah, MD**

Department of Child Health, Kumasi South Hospital Kumasi, Ashanti Region, Ghana

**Professor María Pía Zamarbide, MD**

Departamento de Pediatría, Hospital de Niños Santísima Trinidad, Córdoba, Argentina

## Contributions of Global PARITY Investigator Authors

Data curation, Manuscript review and editing: AA-M, NA, PA, KHA-A, JA, PCA, AA, FA, LYA, JGA, JA, NÁG, TB, BDBH, HBM, JSC-C, MLC, PC, DC, CPC, KESA, TE, MLE, SE, AF, EF, ALF, MG, SGR, MIH, PH, DH, LCHS, NH, SH, JI, SJ, JCJ-B, LPJ, HK, CKK, AMK, M-CFK, NK, GK-L, KK, JDLN, MNM, IMC, ENMN, CIMA, RYM, FM, JHN, KN, MNN, EOO, CEO, ÇÖ, TO, AKO-G, SAO, LDO, MMP, FIP, ALP, WSP, JP, ACR, CRRH, KR, PR, ATR, FS, JS, HRS, JAS, AGS, AMLS, ES, JMS-A, KS, SOS, AVT, AMT, MMT, CU, PV-H, AvSA-vA, JW, RCY, RFY, MPZ.

# Study Collaborators

Collaborators are individuals who made a significant contribution to at least one of the following areas: the conception or design of the work; or the acquisition, analysis, or interpretation of data; or drafting the work or revising it critically for important intellectual content. We would like to acknowledge the many collaborators across participating sites who assisted in making this study possible.

| \| Sophia \| Agomuo \| \| --- \| --- \| \| Rosemary \| Akuaku \| \| Mekdes Shifeta \| Argaw \| \| Fehmina \| Arif \| \| Emmanuel \| Ayingayure \| \| Teena \| Bai \| \| Faisal \| Balouch \| \| Fathea \| Bani \| \| Martin \| Cañon \| \| Efrén Esteban \| Cerón Muñoz \| \| Kwadwo Apeado \| Danso \| \| Bright Richard \| Danyoh \| \| Faisal \| Doud \| \| Milton Henschel \| Ewusi \| \| Khatija \| Farooq \| \| Halwan Yaninga \| Fuseini \| \| Edelweis \| Garavaglia \| \| Tomas \| Gimenez \| \| Kingsley \| Hattoh \| \| Zunaira \| Ibrahim \| \| Kanika \| Jaravta \| \| Mohammed Hafiz \| Kanamu \| \| Sidra \| Khan \| \| Erika \| Miller \| \| Victoria \| Mlele \| \| Zoya \| Mustafa \| \| Grace \| Opoku \| \| Otema \| Owusu-Ansah \| \| Leandro Javier \| Pastori \| \| Sofia \| Piantanida \| \| Sandesha \| Raj \| \| Sibiry \| Samate \| \| Doh \| Sanogo \| \| Emnet Tesfaye \| Shimber \| \| Kathleen \| Sun \| \| Janine \| Taitt \| \| Aminu \| Wada \| \| Haroon \| Zaman \| |  |
| --- | --- | --- | --- | --- | --- | --- | --- | --- | --- | --- | --- | --- | --- | --- | --- | --- | --- | --- | --- | --- | --- | --- | --- | --- | --- | --- | --- | --- | --- | --- | --- | --- | --- | --- | --- | --- | --- | --- | --- | --- | --- | --- | --- | --- | --- | --- | --- | --- | --- | --- | --- | --- | --- | --- | --- | --- | --- | --- | --- | --- | --- | --- | --- | --- | --- | --- | --- | --- | --- | --- | --- | --- | --- | --- | --- | --- | --- |

# References

1. Emeriaud G, Lopez-Fernandez YM, Iyer NP, et al. Executive Summary of the Second International Guidelines for the Diagnosis and Management of Pediatric Acute Respiratory Distress Syndrome (PALICC-2). *Pediatr Crit Care Med*. Feb 1 2023;24(2):143-168. doi:10.1097/PCC.0000000000003147
